# LSD1 inhibition attenuates targeted therapy-induced lineage plasticity in *BRAF* mutant colorectal cancer

**DOI:** 10.1186/s12943-025-02311-z

**Published:** 2025-04-23

**Authors:** Christopher A. Ladaika, Averi Chakraborty, Ashiq Masood, Galen Hostetter, Joo Mi Yi, Heather M. O’Hagan

**Affiliations:** 1 Genome, Cell, and Developmental Biology Graduate Program, Department of Biology, Indiana University Bloomington, Bloomington, IN 47405 USA; 2 Medical Sciences Program, Indiana University School of Medicine, Bloomington, IN 47405 USA; 3 Indiana University Melvin and Bren Simon Comprehensive Cancer Center, Indianapolis, IN 46202 USA; 4 Cell, Molecular and Cancer Biology Graduate Program, Indiana University School of Medicine, Bloomington, IN 47405 USA; 5 Department of Medicine, Indiana University School of Medicine, Indianapolis, IN 46202 USA; 6 Pathology and Biorepository Core, Van Andel Institute, Grand Rapids, MI 49503 USA; 7 Department of Microbiology and Immunology, College of Medicine, Inje University, Busan, South Korea; 8 Department of Medical and Molecular Genetics, Indiana University School of Medicine, Indianapolis, IN 46202 USA

**Keywords:** Colorectal cancer, Lineage plasticity, Neuroendocrine, LSD1, BRAF, Encorafenib, Cetuximab

## Abstract

**Background:**

*BRAF* activating mutations occur in approximately 10% of metastatic colorectal cancer (CRCs) and are associated with worse prognosis in part due to an inferior response to standard chemotherapy. Standard of care for patients with refractory metastatic *BRAF*^*V600E*^ CRC is treatment with BRAF and EGFR inhibitors and recent FDA approval was given to use these inhibitors in combination with chemotherapy for patients with treatment naïve metastatic *BRAF*^*V600E*^ CRC. Lineage plasticity to neuroendocrine cancer is an emerging mechanism of targeted therapy resistance in several cancer types. Enteroendocrine cells (EECs), the neuroendocrine cell of the intestine, are uniquely present in *BRAF* mutant CRC as compared to *BRAF* wildtype CRC.

**Methods:**

BRAF plus EGFR inhibitor treatment induced changes in cell composition were determined by gene expression, imaging and single cell approaches in multiple models of *BRAF* mutant CRC. Furthermore, multiple clinically relevant inhibitors of the lysine demethylase LSD1 were tested to determine which inhibitor blocked the changes in cell composition.

**Results:**

Combined BRAF and EGFR inhibition enriched for EECs in all *BRAF* mutant CRC models tested. Additionally, EECs and other secretory cell types were enriched in a subset of *BRAF*^*V600E*^ CRC patient samples following targeted therapy. Importantly, inhibition of LSD1 with a clinically relevant inhibitor attenuated targeted therapy-induced EEC enrichment through blocking the interaction of LSD1, CoREST2 and STAT3.

**Conclusions:**

Our findings that BRAF plus EGFR inhibition induces lineage plasticity in *BRAF*^*V600E*^ CRC represents a new paradigm for how resistance to BRAF plus EGFR inhibition occurs. Additionally, our finding that LSD1 inhibition blocks lineage plasticity has the potential to improve responses to BRAF plus EGFR inhibitor therapy in patients.

**Supplementary Information:**

The online version contains supplementary material available at 10.1186/s12943-025-02311-z.

## Background

*BRAF* activating mutations occur in approximately 10% of metastatic colorectal cancers (CRCs) and are associated with poor prognosis in part due to an inferior response to high-dose 5-Flurouracil-based chemotherapy [1]. *BRAF*^*V600E*^ mutation activates mitogen-activate protein kinase (MAPK) signaling, a pathway that has been a significant focus for targeted therapy approaches. Findings from the BEACON (Binimetinib, Encorafenib, And Cetuximab cOmbiNed) clinical trial resulted in the standard of care for patients with refractory metastatic *BRAF*^*V600E*^ CRC becoming combined BRAF (encorafenib) and epidermal growth factor receptor (EGFR; cetuximab) inhibition [2]. However, the median overall survival of patients receiving combined therapy is only 9 months. Due the results of the BREAKWATER trial, the FDA recently gave accelerated approval for the use of encorafenib with cetuximab and mFOLFOX6 (5-FU, leucovorin, oxaliplatin) in patients with treatment naïve metastatic *BRAF*^*V600E*^ CRC increasing the patient population who will be receiving BRAF and EGFR inhibitor therapy [3].

Targeted therapies have been developed for many cancers to inactivate mutated or hyperactive proteins that drive cancer-promoting signaling pathways. However, cancers have developed methods of resistance to recent potent targeted therapies that include cancers undergoing shifts in cell identity resulting in cancer cells that are no longer reliant on the signaling pathway being targeted [4]. For example, transformation from adenocarcinoma to neuroendocrine prostate cancer is a mechanism of resistance to treatment with androgen receptor signaling inhibitors in about 20% of castrate-resistant prostate cancer [5]. Additionally, treatment of non-small cell lung cancer (NSCLC) with EGFRi induces transdifferentiation to SCLC, which has a poorly differentiated neuroendocrine phenotype that is correlated with rapid growth and a high metastatic rate [6].

The normal intestinal epithelium contains multiple cell types, including absorptive enterocytes and secretory goblet and enteroendocrine cells (EECs) [7]. Factors released by secretory cells have roles in the normal colon that promote wound healing, gut integrity, and immune cell regulation but can also be tumor promoting [8]. Furthermore, secretory progenitors and EECs are capable of reverting to stem cells after crypt damage [9, 10], which gives these cells the capability of promoting tumor survival under stressful conditions such as during metastasis or treatment. We have demonstrated that EECs and goblet cells are enriched in *BRAF* mutant CRC and promote cancer cell survival via secreted factors [11]. LSD1 (KDM1A), a lysine demethylase overexpressed in CRC, has been implicated in fate specification of multiple cellular lineages in normal and cancerous cells [12]. We have demonstrated that LSD1 is a major regulator of EEC differentiation in *BRAF*^*V600E*^ CRC through interacting with CoREST2 and potentiating STAT3 activity [11, 13].

In the normal colon, MAPK pathway inhibition pushes secretory cell differentiation toward the EEC lineage [14]. Therefore, we sought to determine how standard of care treatment for metastatic *BRAF*^*V600E*^ CRC, which targets the MAPK pathway, alters cancer epithelial cell types in *BRAF*^*V600E*^ CRC. We determined that combined BRAFi plus EGFRi specifically enriched for EECs in several cell line, organoid and colon orthotopic in vivo models of *BRAF* mutant CRC. Furthermore, we demonstrated that LSD1 promoted tumor heterogeneity and lineage plasticity in *BRAF*^*V600E*^ CRC and that targeting LSD1 by epigenetic therapy improved responses to BRAFi plus EGFRi by blocking therapy-induced lineage plasticity.

## Methods

### Cell line and organoid growth and treatment

All cell lines were cultured and maintained as described in [13]. All cell lines were purchased from ATCC. HT29 and NCI-H508 cells were authenticated and last tested for Mycoplasma using the Universal mycoplasma detection kit (ATCC, 30–1012 K) in 2023. All cells used in experiments were passaged fewer than 15 times with most being passaged fewer than 10 times. 817,829 284-R (817) colon cancer organoids were derived from a patient derived xenograft model obtained from the NCI Patient-Derived Models Repository and passaged and differentiated as described previously [11]. Mouse TP KO (Tgfbr2 Trp53 knockout) CRC organoids were generated and passaged as described in the supplementary methods. Encorafenib (MedChemExpress #HY-15605), gefitinib (MedChemExpress #HY-50895), Stattic (Selleckchem #S7024), and SP-2577 mesylate (Seclidemstat mesylate; MedChemExpress #HY-103713 A) were solubilized in DMSO (VWR #97063-136) prior to treatment. Pharmaceutical grade cetuximab was used for in vitro and in vivo experiments.

### RNA isolation and RT-qPCR

RNA isolation and RT-qPCR was performed as in Ladaika et al. [13] and using primers and TaqMan assays listed in Supplementary Table S4. Cq values of genes of interest were normalized to housekeeping gene expression and then to the DMSO control.

### Immunofluorescence

Immunofluorescence for βIII-Tubulin was performed as described in Ladaika et al. [13] using antibodies listed in Supplementary Table S5.

### Dose response curves

Dose response curves were performed by performing serial dilutions of media containing encorafenib or gefitinib into media containing DMSO, gefitinib, encorafenib and/or LSD1 inhibitors as indicated in figure legends. Following 72 H of treatment, cell viability was assayed using CellTiter-Glo Luminescent Cell Viability Assay (Promega #G7572) per manufacturer’s protocol.

### Co-immunoprecipitation

Co-immunoprecipitations from nuclear lysates, chromatin fractionation, and western blots were performed as in Ladaika et al. [13] using antibodies listed in Supplementary Table S5. All western blot images in figures have been cropped. Uncropped images of western blots are included in Additional File 5.

### Orthoptic implantation

All mouse experiments were covered under a protocol approved by the Indiana University Bloomington Animal Care and Use Committee in accordance with the Association for Assessment and Accreditation of Laboratory Animal Care International. Approximately 2.0 × 10^5 HT29 cells expressing firefly luciferase and TdTomato (HT29 LUC2) or dissociated 817 organoids were combined with Matrigel and injected into the mechanically prolapsed large intestines of 6-week-old female NSG mice in a manner similar to what has been done previously [15]. Briefly, mice were anaesthetized by isoflurane inhalation, injected i.p. with buprenorphine, and placed in a supine position with extremities secured to a platform. A blunt-ended hemostat (Micro-Mosquito, 13010-12, Fine Science Tools) was inserted approximately 1 cm into the anus, angled towards the mucosae and opened slightly so that a single mucosal fold was clasped by closing the hemostat to the first notch. The hemostat was then retracted from the anus, exposing the clasped exteriorized mucosae. A 20 µl sterile suspension of cells/partial organoids mixed with 50% sterile Matrigel (Corning) was injected directly into the colonic mucosae. Following tumor formation, mice were treated with BRAFi (encorafenib, 20 mg kg^− 1^, daily, oral gavage) and EGFRi (cetuximab, 20 mg kg^− 1^, biweekly, i.p.) with or without LSD1i (sp-2577 mesylate, 150 mg kg^− 1^, twice daily, oral gavage, as in [16]) for three weeks. Two mice in the BRAFi + EGFRi + SP-2577 reached humane endpoints at 7 days post treatment and two mice from each treatment group were euthanized at this time point for comparison. The remaining three mice per group were treated for a total of 3 weeks. For syngeneic tumors, dissociated TP KO organoids expressing luciferase were combined with Matrigel and injected into the mechanically prolapsed large intestines of 4–6-week-old male and female C57Bl/6 mice. Following tumor formation, mice were treated with BRAFi (encorafenib, 20 mg kg^− 1^, daily, oral gavage) and EGFRi (gefitinib, 100 mg kg^− 1^, daily, oral gavage) for 21 days. Tumors were measured and tissue was assayed as indicated in the supplementary methods. The maximum allowable tumor size of 2000 mm^3^ was not exceeded in any experiment.

### Immunohistochemistry

Immunohistochemistry was performed as in Ladaika et al. [13] using antibodies listed in Supplementary Table S5. Staining percentage was scored blindly using ImageJ for color deconvolution and to determine the percent DAB staining of the total tumor area. Tumor grade was evaluated by standard criteria in human tumors with Grade 1,2 regions defined by > 50% glandular features and Grades 3, 4 defined by decreased glandular features and increased solid and cord-like structures. All cases were stained by H&E and scanned by Aperio with digital files annotated to include the entire tumor mass in semi-quantitative method with outlining of all tumor; low grade tumor; and high grade tumor by board certified pathologist (G. Hostetter) and overall tumor composition based on total tumor area (um2) and the area subset grade 3,4.

### Chromium single cell flex gene expression

#### Sample preparation, library preparation and sequencing

For sample preparation, 20–25 mg of fresh TP KO tumors (7 vehicle and 8 BRAFi + EGFRi) or 817 (3 vehicle and 3 BRAFi + EGFRi) were processed using the Tissue Fixation and Dissociation for Chromium Fixed RNA Profiling protocol (CG000553, 10X Genomics) and the Chromium Next GEM Single Cell Fixed RNA Sample Preparation Kit (1000414, 10X Genomics). See supplementary methods for additional details. Gene expression libraries were prepared at the Indiana University School of Medicine (IUSM) Center for Medical Genomics using the Chromium Fixed RNA Kit, Mouse Transcriptome (1000497, 10x Genomics), in accordance with the user guide (Chromium Fixed RNA Profiling, CG000527- RevD). Briefly, 8,000 cells per sample were targeted for Gel-Beads-in-Emulsion (GEMs) formation after hybridization and pooling equally. The final library was generated and then sequenced using an Illumina NovaSeq X plus with approximately 2500 million reads.

#### Pre-processing and QC

Read alignment and gene quantification of singe cell RNA sequencing (scRNA-seq) data was done using the CellRanger multi pipeline (version 8.0.1, 10X Genomics; Pleasanton, CA, USA). For data from TP KO tumors, the pre-built Cell Ranger mouse reference package (mm10) and mouse reference probe set (Chromium Mouse Transcriptome Probe Set v1.0.1) were used for read alignment. For data from 817 tumors, the pre-built Cell Ranger human reference package (GRCh38-2024) and human reference probe set (Chromium Human Transcriptome Probe Set v1.0.1) were used for read alignment. Additional QC and preprocessing steps are in the Supplementary Methods.

#### Subsetting of tumor epithelial cells and visualizing data

To identify tumor epithelial cells in the dataset, cells were clustered using the functions *FindNeighbors* and *FindClusters*. For the TP KO data. tumor epithelial cells were distinguished from non-epithelial cells using expression levels of epithelial markers and isolated from the Seurat object using the *subset* function. The resulting Seurat object was then normalized, variable features were identified, and the data was scaled, prior to identifying clusters using the *FindNeighbors and FindClusters* function. For the 817 data, human probes were used but some probes likely bound to RNA from mouse cells present in the tumor microenvironment. Therefore, four clusters were removed from the analysis based on marker gene expression that indicated they were likely mouse stromal and immune cells. Cell clusters were then annotated based on marker analysis. Cell proportion changes were calculated using the *propeller* function from the *speckle* package v1.2.0 [17].

#### Analysis of patient ScRNAseq data

Filtered matrices and accompanying metadata obtained from the Broad Institute (see Data Availability Statement) were used to make a Seurat object. Similar pre-processing and quality control steps were taken as indicated for the mouse Flex scRNAseq experiment. Prior to clustering, cells were split into separate Seurat objects based on patient identification and treatment status. These Seurat objects were then integrated together using *FindIntegrationAnchors and IntegrateData.* Samples were then renormalized, scaled, and PCA reduction was performed. Clustering and module scores were generated as described earlier. Significance in cell proportion changes were generated using *scProportion* [11].

## Results

### Combined BRAF plus EGFR inhibition enriches for EECs in *BRAF* mutant CRC

**Fig. 1:**
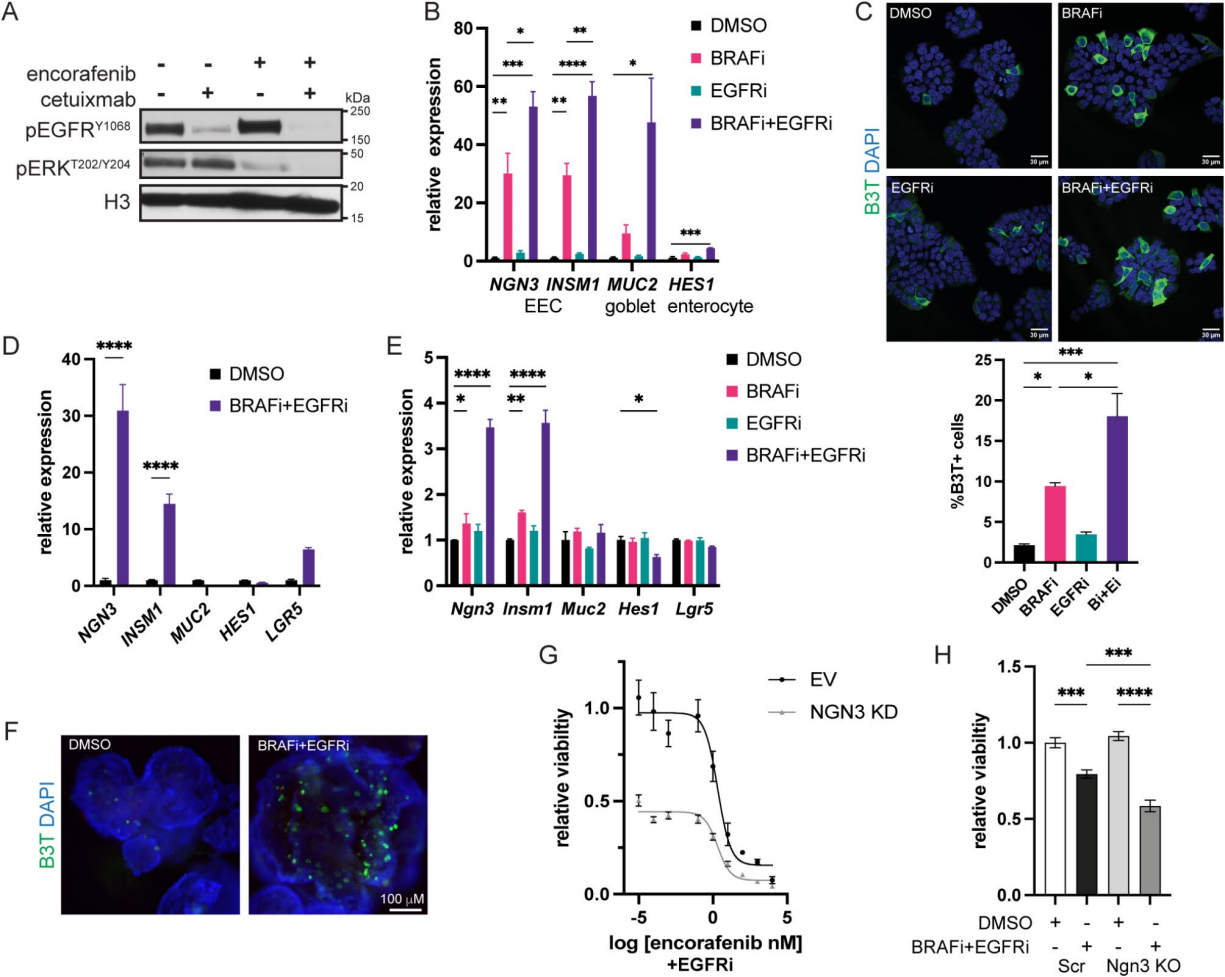
BRAF plus EGFR inhibition enriches for EECs in *BRAF*^*V600E*^ CRC. (**A**) Western blots of HT29 cells treated with DMSO or 2.5 nM encorafenib (BRAFi, Bi) alone or in combination with 20 µg/ml cetuximab (EGFRi, Ei) for 48 H. (**B**) Relative gene expression of indicated genes in HT29 cells treated as in (**A**) Gene expression was normalized to the housekeeping gene *RHOA* and then to the DMSO treated cells. Graph represents mean +/- SEM. *N* = 3. (**C**) Immunofluorescence for β3-tubulin (B3T) in HT29 cells treated as in A for 72 H. Graph (below) is the %b3-tubulin positive cells of the total number of cells per field. *N* = 3. (**D**) Relative gene expression of indicated genes in 817 human CRC organoids treated with DMSO or 2.5 nM encorafenib (BRAFi) and 500 nM gefitinib (EGFRi) for 6 days. Data is normalized and presented as in (**B**) (**E**) Relative gene expression of indicated genes in TP KO mouse CRC organoids treated with DMSO or 2.5 nM encorafenib (BRAFi) alone or in combination with 500 nM gefitinib (EGFRi) for 4 days. Data is normalized and presented as in B. (**F**) Immunofluorescence for β3-tubulin (B3T) in TP KO organoids treated as in E. (**G**) Encorafenib dose response curve of empty vector (EV) or NGN3 knockdown (KD) HT29 cells treated with 500 nM gefitinib (EGFRi) for 72 H. Viability was normalized to DMSO treated EV cells. *N* = 3. (**H**) Relative viability of TP KO scramble (Scr) and NGN3 KO organoids treated as in E. Viability was normalized to DMSO treated scramble cells. *N* = 3. Significance was determined by one-way ANOVA with Tukey pairwise multiple comparison testing. *P ≤ 0.05, ** *P* ≤ 0.01, *** *P* ≤ 0.001, **** *P* ≤ 0.0001

As targeted therapies have been shown to induce cell plasticity in other cancer types [4], we treated the *BRAF*^*V600E*^ mutant CRC cell line HT29 with BRAFi (encorafenib) and EGFRi (cetuximab or gefitnib) alone or in combination. Individual treatments of EGFRi and BRAFi reduced levels of phosphorylated EGFR (pEGFR) and ERK (pERK), respectively, and combined BRAFi + EGFRi reduced pERK levels further than BRAFi alone (Fig. 1A, S1A). Interestingly, BRAFi alone or in combination with EGFRi increased expression of EEC marker genes *NGN3* and *INSM1* but had less of an effect on the expression of marker genes of goblet cells (*MUC2*) or enterocytes (*HES1*) (Fig. 1B, S1B). As EEC marker gene expression increased with length of treatment and doses of encorafenib between 1 and 100 nM had similar effects on expression of EEC genes, most future experiments were performed at 2.5 nM encorafenib (Fig. S1C, S1D). Consistent with the gene expression findings, BRAFi or BRAFi + EGFRi increased the percentage of cells positive for βIII-3-tubulin, a marker of neuronal cells that is also expressed in EECs (Fig. 1C, S1E). BRAFi + EGFRi also increased expression of EEC marker genes *NGN3* and *INSM1* in a *BRAF*^*V600E*^ CRC patient derived human organoid model 817 without changing expression of goblet or enterocyte markers (Fig. 1D). As MEK inhibitors (MEKi) have also been used to treat patients with metastatic *BRAF*^*V600E*^ CRC, we tested the effect of MEKi on marker gene expression in HT29 cells. Increasing concentrations of MEKi alone increased expression of EEC genes *NGN3* and *INSM1* and goblet gene *MUC2* and, to a lesser extent, enterocyte gene *HES1* (Fig. S1F). MEKi alone or in combination with EGFRi also increased the percentage of β3-tubulin positive cells (Fig. S1G). NCI-H508 CRC cells have a class 3 BRAF mutation, G596R, and patients with this mutation are not treated with encorafenib [18]. However, because the NCI-H508 cell line contains goblet cells and EECs [11, 13] we were interested in how MAPK inhibition would impact EEC differentiation in these cells. BRAFi treatment alone had no effect on pERK, EGFRi had a modest effect on pEGFR and reduced pERK levels, and the reduction in pERK and/or pEGFR was greatest when EGFRi was used in combination with BRAFi (Fig. S1H). EGFRi alone or in combination with BRAFi increased expression of EEC marker genes and the percentage of β3-tubulin positive cells in NCI-H508 cells (Fig. S1I, S1J, S1K).

To generate a model of metastatic *BRAF*^*V600E*^ CRC to use in vitro and in immunocompetent mice, we derived tumor organoids from tumors from an in situ model of *BRAF*^*V600E*^*Apc*^*D716/+*^ tumorigenesis [19, 20]. Because we were interested in ultimately studying metastatic CRC, we then knocked out Tgfbr2, which has reduced expression in *BRAF* mutant CRC as compared to normal colon and *BRAF* wildtype CRC, and Trp53 in the organoids using a CRISPR/Cas9 approach (Fig. S1L, S1M). Interestingly, the Tgbfr2 + Trp53 knockout (TP KO) organoids had reduced expression of Trp53 target gene *Bax1*, and tumor suppressor gene *Rb1*, as well as increased expression of EEC marker genes, *Ngn3* and *Insm1*, and stem cell gene *Lgr5* relative to the mock KO organoids (Fig. S1N, S1O). Single agent treatment only had modest effects on expression of marker genes in the TP KO organoids, whereas combined BRAFi + EGFRi treatment significantly increased EEC marker gene expression (Fig. 1E, S1P). Immunofluorescence for b3-tubulin confirmed that BRAFi + EGFRi treatment increased the percentage of EECs in the TP KO organoids (Fig. 1F).

The transcription factor NGN3 (NEUROG3) is required for the differentiation of EECs in the intestine [21, 22]. Therefore, to determine if the presence of EECs influences the sensitivity of the cells to treatment, we knocked down NGN3 in HT29 cells (S1Q) and performed an encorafenib dose response curve in the presence of a constant concentration of gefitinib. NGN3 knockdown (KD) cells had decreased viability relative to empty vector (EV) control KD cells and were more sensitive to encorafenib treatment (Fig. 1G). We also knocked out Ngn3 in the TP KO organoids to reduce EECs (Fig. R, S1S). While Ngn3 KO had no effect on the baseline viability of the TP KO organoids, BRAFi + EGFRi reduced viability of the Ngn3 KO organoids to a greater extent than the scramble control organoids (Fig. 1H). Altogether, these findings demonstrate that MAPK inhibition achieved by BRAFi and/or EGFRi treatment enriches for EECs in *BRAF* mutant CRC and suggests that EECs promote resistance to this therapy.

### Residual tumors following BRAF plus EGFR inhibitor treatment have altered tumor cell composition

**Fig. 2:**
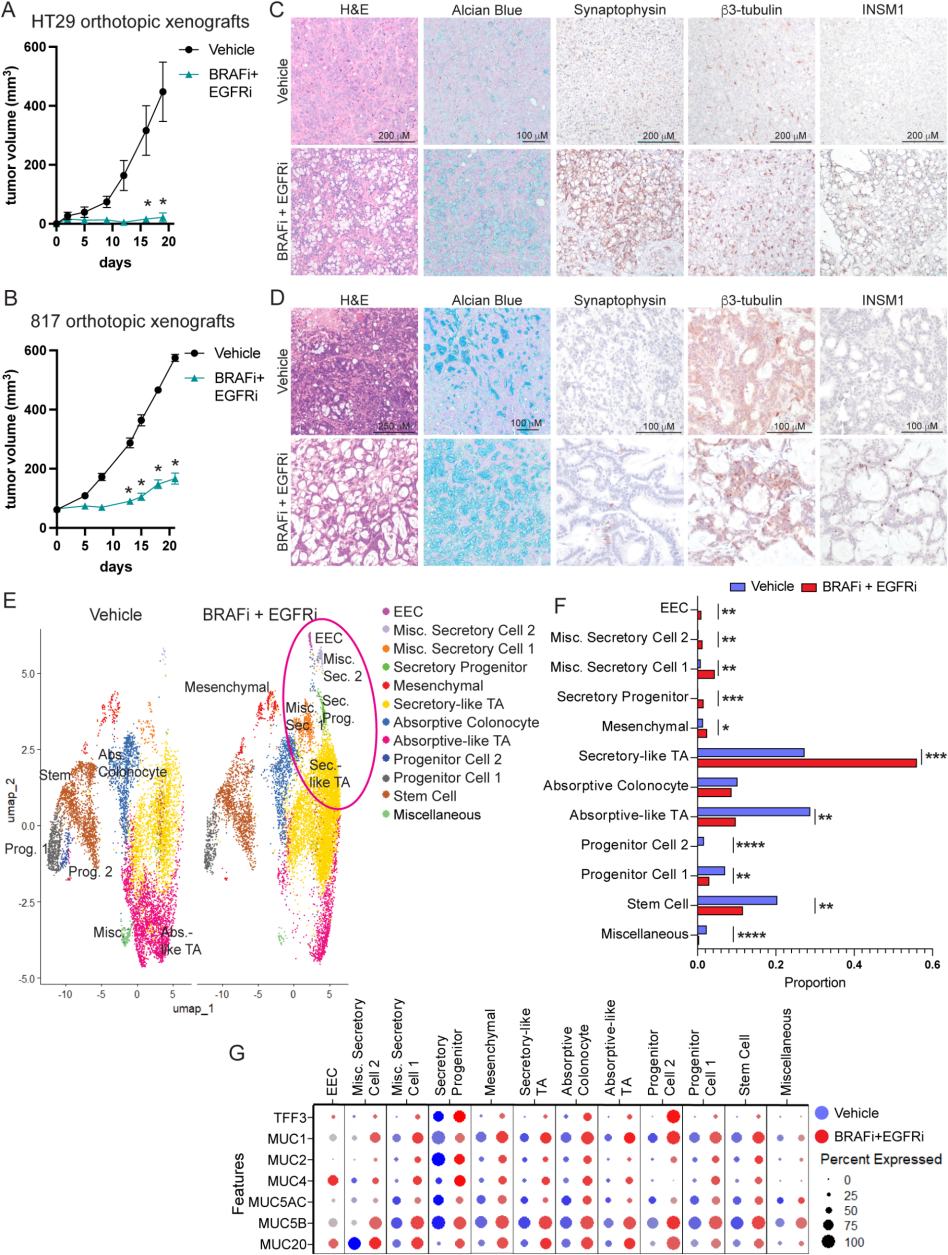
BRAFi plus EGFRi treatment induces a neuroendocrine phenotype in residual *BRAF*^*V600E*^ tumors. (**A**) Following tumor formation of HT29 cells or (**B**) 817 human CRC organoids orthotopically engrafted into the colons of NSG mice, mice were treated with vehicle or BRAFi (encorafenib, 20 mg kg^− 1^, daily) and EGFRi (cetuximab, 20 mg kg^− 1^, biweekly). Graph is the mean +/- SEM of the tumor volume measured by caliper over the course of treatment. *N* = 5 mice per group. (**C**) Representative H&E, Alcian blue and IHC images of HT29 colon tumors from the experiment in (**A**) (**D**) Representative H&E, Alcian blue and IHC images of 817 colon tumors from the experiment in (**B**) (**E**) UMAP dot plot of 817 tumor scRNAseq data from mice treated with vehicle (left) or BRAFi + EGFRi (right). Samples are colored by cell type/cluster. TAs are transit amplifying cells, EECs are enteroendocrine cells and misc. stands for miscellaneous. Secretory populations increased in BRAFi + EGFRi tumors are circled in pink. (**F**) Fold change in cell type proportions in BRAFi + EGFRi relative to vehicle samples. (**G**) Dot plot showing mucous related gene expression in vehicle and BRAFi + EGFRi treated samples across all annotated cell types. The size of the dot is proportional to the percentage of cells that express a given gene, and the color scale indicates the average scaled gene expression within the specific cell population. Blue and red dots are expression levels in cells from vehicle and BRAFi + EGFRi tumors, respectively. Significance was determined by two-way ANOVA corrected for multiple comparisons using the Šídák method (**A, B**) and by a moderated t-test (**F**). *P ≤ 0.05, ** *P* ≤ 0.01, *** *P* ≤ 0.001, **** *P* ≤ 0.0001

To determine how BRAFi + EGFRi treatment alters tumor composition in vivo, we orthotopically engrafted HT29 cells expressing tdTomato and luciferase or 817 organoids into the colons of NSG mice (Fig. S2A). After tumor formation, mice were treated for 3 weeks with encorafenib (BRAFi) and cetuximab (EGFRi). The treatment prevented and reduced growth of HT29 and 817 orthotopic tumors, respectively (Fig. 2A and B), and significantly reduced lung metastasis of both tumor types (Fig. S2B). Treatment reduced levels of phosphorylated ERK (pERK) as expected and reduced Ki67 staining without increasing the apoptosis marker, cleaved caspase 3 (Fig. S2C, S2D). Interestingly, by H&E staining, all residual tumors had increased intratumoral cystic spaces, which were mostly positive for Alcian blue stain, an indicator of acidic mucins (Fig. 2C and D). Consistent with our in vitro data, the residual HT29 tumors from treated mice were enriched for EECs as indicated by increased synaptophysin, β3-tubulin, and INSM1 staining (Fig. 2C, S2E). Synaptophysin, β3-tubulin and INSM1 positive cells were also increased in 817 tumors from BRAFi + EGFRi compared to vehicle treated mice but, unlike in the HT29 tumors, the positive cells were limited to small pockets of cells (Fig. 2D).

As the EEC enrichment was not as dramatic in the 817 tumors as compared to the HT29 tumors (Fig. 2C and D), we wanted to determine how BRAFi + EGFRi altered tumor composition of the 817 tumors using an unbiased approach. Therefore, we performed FLEX single cell RNA-sequencing (scRNA-seq) on 817 tumors from three vehicle and three BRAFi + EGFRi treated mice. Clusters were defined by cell cycle profile, trajectory analysis and expression of marker genes (Fig. S2F, S2G, S2H, Supplementary Table S1). Interestingly, the proportions of several clusters of cells with secretory-like gene expression were higher in the BRAFi + EGFRi tumors than vehicle tumors, including EECs, secretory progenitors, mesenchymal cells, and secretory-like TA cells (Fig. 2F). Vehicle tumors had higher proportions of absorptive, progenitor and stem cell populations. One of the strongest marker genes for the progenitor 2, absorptive-like TA and miscellaneous cell populations that were enriched in the vehicle treated tumors was *NDGR1* (N-myc downstream-regulated gene 1), which is a stress response protein induced by hypoxia and connected to cetuximab sensitivity in CRC (Fig. S2I) [23, 24]. These clusters are also enriched for the Hallmark hypoxia gene set (Fig. S2J). Even though the 817 tumors all had pockets of mucous (Fig. 2D), we did not identify a goblet cell cluster in the scRNA-seq data. Instead, most of the cell clusters expressed several mucins and mucin-related genes, with the expression of these genes increasing with treatment in some clusters (Fig. 2G).

**Fig. 3:**
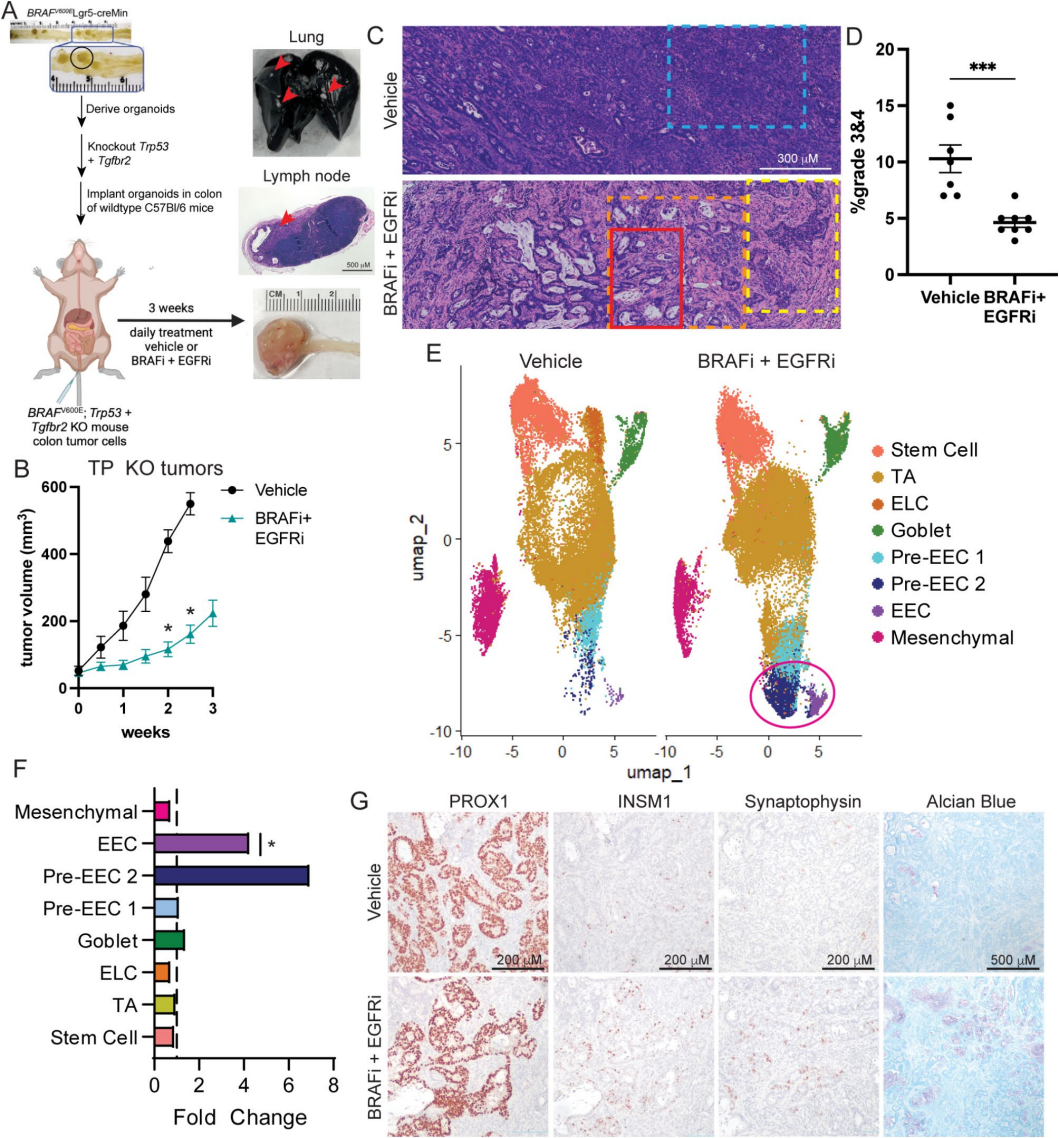
BRAFi plus EGFRi treatment induces a neuroendocrine phenotype in a syngeneic model of *BRAF*^*V600E*^ CRC. (**A**) Diagram for the generation of TP KO organoids and TP KO colon orthoptic model and treatment, including representative images of a colon tumor and lung and lymph node metastases (indicated by red arrows). (**B**) Following tumor formation of TP KO mouse CRC organoids orthotopically engrafted into the colons of C57Bl/6 mice, mice were treated with vehicle or BRAFi (encorafenib, 20 mg kg^− 1^, daily) and EGFRi (gefitinib, 75 mg kg^− 1^, daily). Graph is the mean +/- SEM of the tumor volume measured by caliper over the course of treatment. *N* = 7, vehicle. *N* = 8, BRAFi + EGFRi. (**C**) Representative H&E images of TP KO tumors. Blue dashed box indicates undifferentiated tumor glands, grade 3,4 solid and cord like tumor with dense stroma. Red solid box indicates well differentiated glandular tumor with goblet cells and active mucin production. Orange dashed box indicates region with well-defined mature stroma. Yellow dashed box indicates less differentiated solid tumor area with biphasic appearance suggestive of neuroendocrine features. (**D**) Semi quantitative percentage of TP KO tumor areas that are grade 3&4. Each point represents the scoring for one tumor. Lines represent mean -/+ SEM. (**E**) UMAP dot plot of TP KO tumor epithelial cell scRNAseq data from mice treated with vehicle (left) or BRAFi + EGFRi (right). Samples are colored by cell type/cluster. TAs are transit amplifying cells, ELCs are enterocyte-like cells, and EECs are enteroendocrine cells. EEC populations enriched in BRAFi + EGFRi treated tumors are circled in pink. (**F**) Fold change in cell type proportions in BRAFi + EGFRi relative to vehicle samples. (**G**) Representative IHC and Alcian blue images of TP KO colon tumors from the experiment in B. Significance was determined by two-way ANOVA corrected for multiple comparisons using the Šídák method (**B**), student’s T-test (**D**) and by a moderated t-test (**F**). *P ≤ 0.05

To explore how BRAFi + EGFRi therapy alters tumor composition in a syngeneic model of *BRAF*^*V600E*^ CRC, we implanted TP KO organoids expressing luciferase into the colons of wildtype C57Bl/6 mice (Fig. 3A). As cetuximab is an anti-human EGFR antibody it lacks activity on mouse EGFR. Therefore, we treated tumor bearing C57Bl/6 mice with encorafenib and the EGFR tyrosine kinase inhibitor gefitinib daily for 3 weeks. Treatment reduced tumor growth and metastasis to the lungs and iliac and lumbar lymph nodes (Fig. 3B, S3A, S3B). Microscopic review revealed that all orthotopic tumors contained regions of mixed histology by tumor grade. The vehicle tumors contained more solid and poorly differentiated regions in contrast to the BRAFi + EGFRi tumors (Fig. 3C and D). In addition, the treated tumors showed significantly increased areas of differentiated glandular structures and were intimately associated with more mature stroma characterized by more ordered, fibrillar and eosinophilia compared to the vehicle tumors (Fig. 3C). To further explore how therapy alters tumor cell composition, we performed FLEX scRNAseq on 7 vehicle and 8 BRAFi + EGFRi tumors. To focus on tumor epithelial cell populations, clustering analysis was performed using only tumor epithelial cells extracted from the scRNAseq data. Clusters were defined by expression of marker genes, cell cycle profile and trajectory analysis (Fig. S3C, S3D, Supplementary Table S2). Furthermore, cluster analysis of module scores for Hallmark gene sets confirmed cluster identification, with stem cells being enriched for MYC and E2F targets, mesenchymal cells being enriched for epithelial to mesenchymal transition (EMT) and TGF beta signaling pathways, and EECs being enriched for pancreas beta cells, a cell type that shares many gene expression pathways with EECs (Fig. S3E). Examining differences in cell type proportions demonstrated significant enrichment of differentiated EECs and a trend towards enrichment of Pre-EEC 2 cells in BRAFi + EGFRi tumors as compared to vehicle tumors (Fig. 3F). Interestingly, *Prox1*, a gene expressed by progenitor cells and EECs in the normal intestine and related to neuroendocrine plasticity in prostate cancer [10, 25], was identified as a marker gene of all pre-EEC and EEC clusters (Fig. S2F). Regions of PROX1 + cells were present in all tumors regardless of treatment with some of the highest positivity occurring in BRAFi + EGFRi tumors (Fig. 3G, S3G). Interestingly, most of the EECs, indicated by INSM1 and synaptophysin IHC, were in PROX1 + areas and the EEC markers were enriched in BRAFi + EGFRi tumors (Fig. 3G, S3G). PROX1 + cells were also present in HT29 and 817 orthotopic tumors and increased in the BRAFi + EGFRi tumors relative to the vehicle tumors (Fig. S3H). Similar to our other models, the cystic regions of the TP KO tumors also stained positively for Alcian blue.

### Inhibition of LSD1 reduces BRAFi + EGFRi-induced cell plasticity

**Fig. 4:**
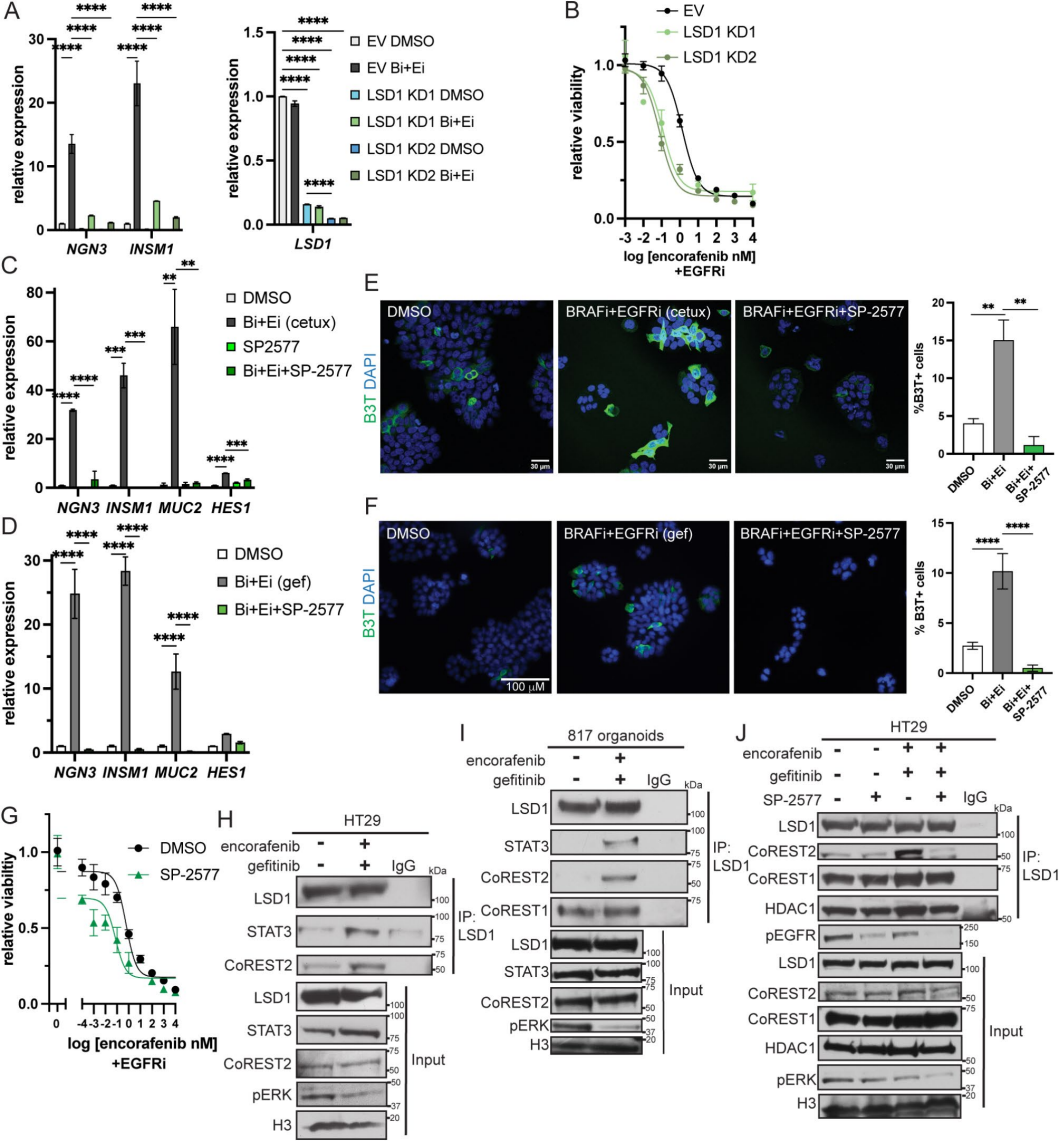
LSD1 inhibition blocks therapy-induced EEC enrichment in *BRAF*^*V600E*^ CRC. (**A**) Relative gene expression in empty vector (EV) and LSD1 knockdown (KD) HT29 cells treated with DMSO or 2.5 nM encorafenib (BRAFi, Bi) plus 500 nM gefitinib (EGFRi, Ei) for 48 H. Gene expression was normalized to the housekeeping gene *RHOA* and then to the DMSO treated cells. Graph represents mean +/- SEM. *N* = 3. LSD1 KD1 and KD2 were generated using different shRNAs. (**B**) Encorafenib dose response curve of EV and LSD1 KD HT29 cells treated with 500 nM gefitinib (EGFRi) for 72 H. Viability was normalized to the respective non-encorafenib treated cells. (**C**) Relative gene expression of indicated genes in HT29 cells treated with DMSO or 2.5 nM encorafenib (BRAFi) and 20 µg/ml cetuximab (EGFRi, Ei, cetux) with or without 1 µM SP-2577 for 48 H. Data presented as in A. (**D**) Relative gene expression of indicated genes in HT29 cells treated with DMSO or 2.5 nM encorafenib (BRAFi, Bi) plus 500 nM gefitinib (EGFRi, Ei, gef) with or without 500 nM SP-2577 (LSD1i) for 4 days. Data presented as in (A) (**E**) Immunofluorescence for β3-tubulin (B3T) in HT29 cells treated as in C for 72 H. Graph is the %β3-tubulin positive cells of the total number of cells per field. *N* = 3. (**F**) Immunofluorescence for β3-tubulin in HT29 cells treated as in D for 72 H and analyzed in E. (**G**) Encorafenib dose response curve of HT29 cells treated with 500 nM gefitinib (EGFRi) and DMSO or 500 nM SP-2577 for 72 H. Data was normalized as in (**B**) (**H**) LSD1 coIP in nuclear lysates prepared from HT29 cells treated with DMSO or 2.5 nM encorafenib plus 500 nM gefitinib for 4 H. (**I**) LSD1 coIP in nuclear lysates prepared from 817 organoids treated with DMSO or 2.5 nM encorafenib plus 500 nM gefitinib for 4 H. (**J**) LSD1 coIP in nuclear lysates prepared from HT29 cells pre-treated with DMSO or 1 µM SP-2577 for 24 H prior to treatment with DMSO or SP-2577 with or without 2.5nM encorafenib and 500 nM gefitinib for 4 H. Significance was determined by one-way ANOVA with Tukey pairwise multiple comparison testing. *P ≤ 0.05, ** *P* ≤ 0.01, *** *P* ≤ 0.001, **** *P* ≤ 0.0001

We had previously demonstrated that LSD1 through its interaction with CoREST2, promotes EEC differentiation in *BRAF*^*V600E*^ CRC [11, 13]. Interestingly, BRAFi alone and BRAFi + EGFRi increased the expression of *RCOR2*, the gene encoding CoREST2, and CoREST2 protein levels but not LSD1 in HT29 cells (Fig. S4A, S4B, S4C). Therefore, we sought to determine if LSD1-CoREST inhibition also blocks therapy-induced EEC enrichment. LSD1 knockdown or inhibition with the LSD1/CoREST dual inhibitor corin reduced the BRAFi + EGFRi and/or BRAFi-induced increase in expression of EEC marker genes in HT29 cells ( [26]; Fig. 4A, S4D, S4E). LSD1 knockdown or inhibition with corin also increased the sensitivity of HT29 cells to BRAFi + EGFRi and/or BRAFi treatment (Fig. 4B, S4F, S4G).To identify a clinically relevant inhibitor to use in our studies, we tested the effect of a panel of LSD1 inhibitors on EEC differentiation [27]. Only SP-2577 (seclidemstat), a noncompetitive reversible LSD1 inhibitor that inhibits the catalytic and scaffolding functions of LSD1 [27], reduced the basal expression of EEC marker genes in HT29 cells even though all the inhibitors reduced global levels of H3K4me2 (Fig. S4H, S4I). SP-2577 also reduced the BRAFi + EGFRi-induced increase in EEC marker gene expression (Fig. 4C and D, S4J, S4K) and increase in β3-tubulin positive EECs (Fig. 4E F, S4L) in HT29 and NCI-H508 cells. Similarly to LSD1 knockdown, SP-2577 increased the sensitivity of HT29 cells to encorafenib treatment and the sensitivity of HT29 and NCI-H508 cells to combined BRAFi + EGFRi treatment (Fig. S4M, 4G, S4N, S4O). SP-2577 treatment also blocked the MEKi-induced increase in expression of EEC marker genes, the MEKi + EGFRi-induced increase in β3-tubulin positive EECs and increased the sensitivity of HT29 cells to MEKi (Fig. S4P, S4Q, S4R).

We recently determined that LSD1 in combination with CoREST2 promotes EEC differentiation by potentiating STAT3 activity [13]. STAT3 signaling has also been shown to promote resistance to targeted therapies, including BRAFi, in CRC [28, 29]. Consistent with our previous findings, STAT3i reduced the BRAFi-induced expression of EEC markers in HT29 cells and BRAFi + EGFRi-induced expression in NCI-H508 cells (Fig. S4S, S4T). BRAFi + EGFRi treatment also induced the interaction of LSD1 with STAT3 and CoREST2 in HT29 and NCI-H508 cells and 817 organoids (Fig. 4H, S4U, 4I). Interestingly, combining SP-2577 with BRAFi + EGFRi treatment blocked the inhibitor-induced increased interaction between LSD1 and CoREST2 in HT29 cells, without effecting the interaction between LSD1 and CoREST1 or HDAC1 (Fig. 4J). SP-2577 also reduced the inhibitor-induced interaction between LSD1 and CoREST2 and STAT3 in NCI-H508 cells (Fig. S4V). LSD1 and CoREST2 demethylate STAT3 to prolong STAT3’s binding to chromatin [13, 30]. Consistent with this role for LSD1/CoREST2, BRAFi + EGFRi increased the binding of STAT3 to chromatin, whereas adding SP-2577 blocked the inhibitor-induced increase in STAT3 chromatin binding (Fig. S4W). Altogether this data, demonstrates that LSD1 is critical for BRAFi + EGFRi-induced EEC differentiation and that SP-2577 blocks BRAFi + EGFRi-induced EEC differentiation likely by disrupting the LSD1-CoREST2-STAT3 interaction.

### LSD1 Inhibition attenuates therapy-induced cell plasticity in vivo

**Fig. 5:**
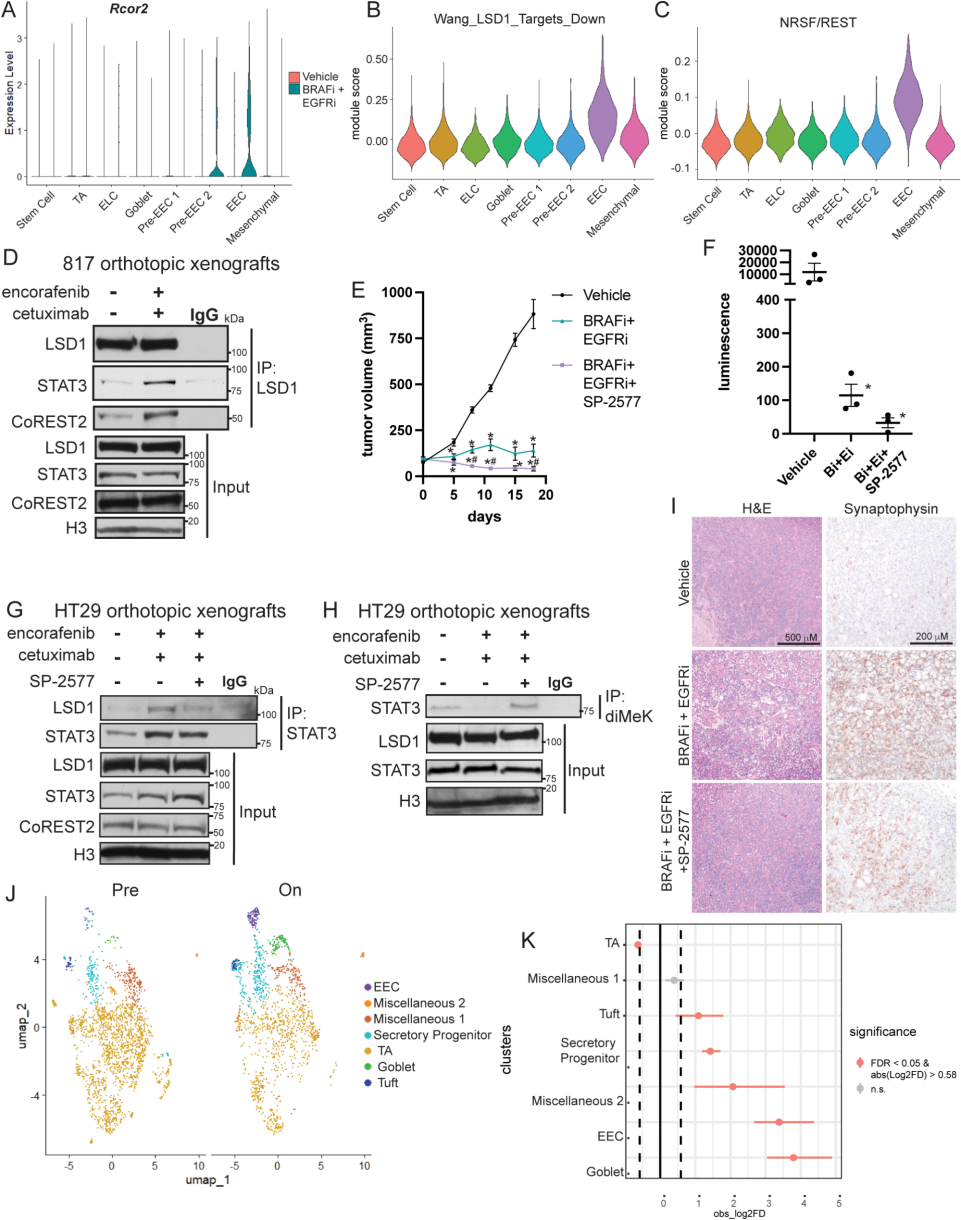
LSD1 inhibition blocks BRAFi + EGFRi-induced lineage plasticity in vivo. (**A**) Rcor2 violin plot from TP KO scRNAseq data. Module scores for the (**B**) Wang LSD1 Targets Down and (**C**) NRSF gene sets for each epithelial cell in the TP KO scRNAseq data. (**D**) LSD1 coIP in nuclear lysates prepared from 817 colon orthotopic tumors from vehicle or encorafenib + cetuximab treated NSG mice. (**E**) Following tumor formation of HT29 cells orthotopically engrafted into the colons of NSG mice, mice were treated with vehicle or BRAFi (encorafenib, 20 mg kg^− 1^, daily) and EGFRi (cetuximab, 20 mg kg^− 1^, biweekly) with or without SP-2577 (LSD1i, 100 mg kg^− 1^, twice daily). Graph is the mean +/- SEM of the tumor volume measured by caliper over the course of treatment. *N* = 5 mice per group at days 0–7. *N* = 3 mice per group at days 12–18. *P ≤ 0.05 relative to vehicle. ^#^*P* ≤ 0.05 relative to BRAFi + EGFRi. Significance was determined by fitting a mixed model and correcting for multiple comparisons by controlling the false discovery rate using the two-stage step up method of Benjamini, Krieger, and Yekutieli. (**F**) Total luminescence signal detected by plate reader from lungs incubated ex vivo in PBS + luciferin. A lung from a non-tumor bearing mouse was used as a negative control. Each point represents an individual lung. Lines represent mean -/+ SEM. *P ≤ 0.05 relative to vehicle. Significance was determined by one-way ANOVA with Tukey pairwise multiple comparison testing. (**G**) STAT3 coIP in nuclear lysates prepared from HT29 colon orthotopic tumors from mice treated as indicated for 7 days. (**H**) Dimethyl lysine IP from nuclear lysates from tumors treated as indicated for 19 days. (**I**) Representative H&E and IHC images of HT29 colon tumors from the mice treated for 19 days from the experiment in E. (**J**) UMAP dot plot of scRNAseq data of pretreated (left) or on treatment (right) samples from patients with *BRAF*^*V600E*^ CRC. Samples are colored by cell type/cluster. TAs are transit amplifying cells and EECs are enteroendocrine cells. (**K**) Relative differences in cell proportions for each cluster between the pretreatment and on treatment samples

We next explored connections between LSD1-CoREST2-STAT3 and EECs in our in vivo models of *BRAF*^*V600E*^ CRC. In our scRNAseq data from TP KO colon orthotopic tumors, *Rcor2*, the gene encoding CoREST2, was predominantly expressed in the Pre-EEC 2 and EEC clusters in BRAFi + EGFRi tumors (Fig. 5A). Additionally, gene set module score analysis demonstrated that EECs were enriched for genes that were down-regulated after LSD1 knockout in the pituitary relative to the other cell populations and for genes that are regulated by repressor element-1 silencing transcription factor/neuron-restrictive silencer factor (REST/NSRF), a repressor of neuroendocrine genes (Fig. 5B and C) [31]. These computational analyses suggested that LSD1-CoREST2 contributed to EEC differentiation in *BRAF*^*V600E*^ CRC following BRAFi + EGFRi treatment in vivo. To further confirm these findings, we performed LSD1 and STAT3 co-IPs from nuclear lysates prepared from HT29 and 817 orthotopic tumors from treated mice. The interaction of LSD1 with both STAT3 and CoREST2 and the interaction of STAT3 with LSD1 was higher in BRAFi + EGFRi than vehicle tumors (Fig. 5D, S5A, S5B).

To determine how LSD1 inhibition altered response to targeted therapy, we implanted HT29 cells orthotopically into the colons of NSG mice and, following tumor formation, treated the mice with vehicle or BRAFi + EGFRi with or without the LSD1i SP-2577. As demonstrated previously, BRAFi + EGFRi treatment reduced tumor growth and levels of phosphorylated ERK and Ki67 (Fig. 5E, S5C, S5D). The addition of SP-2577 further reduced tumor size and Ki67 staining (Fig. 5E, S5C, S5D). Ex vivo luminescence demonstrated that metastasis to the lung was decreased in the BRAFi + EGFRi treated groups, with a trend to further reduction with the addition of SP-2577 (Fig. 5F). In tumor tissue collected after 7 days of treatment, the interaction of STAT3 with LSD1 increased in BRAFi + EGFRi relative to vehicle tumors but this increase was attenuated with the addition of SP-2577 treatment (Fig. 5G). We previously demonstrated that LSD1-CoREST2 demethylates STAT3 to promote STAT3 activity [13]. Here, in tumor lysates prepared from mice treated for three weeks, levels of demethylated STAT3 decreased in BRAFi + EGFRi relative to vehicle tumors but increased in BRAFi + EGFRi + SP-2577 tumors (Fig. 5H). Histologically, the addition of SP-2577 treatment reduced the BRAFi + EGFRi-induced increase in intratumoral cystic areas and Alcian blue and synaptophysin staining (Fig. 5I, S5C, S5E). Collectively, these findings suggest that LSD1 inhibition by SP-2577 treatment reduced the targeted therapy-induced interaction of LSD1-CoREST2-STAT3 and subsequent lineage plasticity in *BRAF*^*V600E*^ CRC.

### Targeted therapy enriches for secretory cells in a subset of patients with *BRAF*^*V600E*^ CRC

To explore if targeted therapy induced lineage plasticity in patients, we analyzed publicly available scRNAseq data from a phase 2 clinical trial where patients with refractory metastatic *BRAF*^*V600E*^ CRC were treated with BRAFi (dabrafenib), MEKi (trametinib) and immunotherapy (anti-PD-1, sparatlizumab) [32]. Pretreatment and day 15 on-treatment scRNAseq data was available for 23 patients. However, samples from only three patients had enough cells in both samples to proceed with analysis (greater than 200) and one of those samples had no expression of EEC marker genes. Therefore, we integrated the scRNAseq data from the two remaining samples and, based on marker analysis, identified clusters representing several types of secretory cells, including secretory progenitors, EECs, goblet cells and tuft cells (Fig. 5J, S5F, Supplementary Table S3). EECs and goblet cell clusters were the most enriched clusters in the on-treatment relative to the pretreatment samples (Fig. 5K). Interestingly, like some of our in vivo models, *PROX1* expression was highest in the secretory cell populations and the number of cells expressing *PROX1* increased in the on-treatment samples (Fig. S5G). Additionally, the EEC cluster showed the greatest enrichment of genes that were downregulated after LSD1 knockout in the pituitary relative to the other cell populations (Fig. S5H). Altogether, these findings suggest that targeted therapy also enriches for secretory cells populations, including EECs, in patients.

## Discussion

Targeted therapy induced lineage plasticity is an emerging mechanism of therapy resistance that has been most well studied in prostate and lung cancer [4]. Here, we demonstrate that *BRAF*^*V600E*^ CRC also undergoes therapy induced lineage plasticity following BRAFi + EGFRi treatment. Neuroendocrine cancers induced by treatment in prostate and lung cancer are highly aggressive, metastatic and therapy resistant, which suggests that lineage plasticity toward a neuroendocrine cancer in *BRAF*^*V600E*^ CRC may contribute to the poor outcomes of patients with this cancer. Recent work has demonstrated that metastatic CRCs are enriched for cells that express alternative lineage gene programs, including neuroendocrine [33], further suggesting that our findings in *BRAF*^*V600E*^ CRC may be connected to the highly metastatic and aggressive nature of this CRC subtype.

As EECs are the neuroendocrine cell of the intestine, it is likely that many pathways contributing to neuroendocrine transformation in prostate and lung cancer are also relevant to *BRAF*^*V600E*^ CRC and vice versa. Additionally, there is existing literature on the role of signaling pathways and other factors in the differentiation of the different types of epithelial cells in the normal intestine that will be informative to therapy induced lineage plasticity in other epithelial cancers. Interestingly, the combination of Wnt and MAPK inhibition in the normal intestine results in EEC differentiation [14]. *BRAF*^*V600E*^ CRC typically has lower Wnt activation than *BRAF* wild type CRC and treatment with BRAFi + EGFRi inhibits MAPK [20]. The resultant state of cell signaling pathway levels may be connected to the enrichment of EECs following BRAFi + EGFRi in *BRAF*^*V600E*^ CRC. However, it is also possible that BRAFi + EGFRi selectively depletes non-EECs in *BRAF*^*V600E*^ CRCs resulting in EEC enrichment. We do not favor this hypothesis, however, because in vivo BRAFi + EGFRi predominantly caused a decrease in proliferation and a change in cell signaling pathway activation, not an increase in cell death.

Epigenetic factors play significant roles in the maintenance of stemness and differentiation pathways. Here, we demonstrate that LSD1-CoREST2 promotes therapy-induced enrichment of EECs in *BRAF*^*V600E*^ CRC. In addition to LSD1, other epigenetic factors have been implicated in lineage plasticity in other cancer types, and it will be of interest to explore them in future studies in CRC [4, 12, 34]. There are several LSD1 inhibitors in clinical trials [12]. However, of the ones we tested, only SP-2577 (seclidemstat) was effective in reducing EEC differentiation. SP-2577 has greater ability to block interactions between LSD1 and other proteins than the other LSD1 inhibitors and has been shown to prevent neuroendocrine differentiation in prostate cancer [16]. We demonstrated that SP-2577 attenuates the inhibitor-induced interaction of LSD1 with CoREST2 and STAT3 and STAT3 chromatin binding, which we have previously shown are required for EEC differentiation [13]. Therefore, it is likely that SP-2577 is effective in our models because of its ability to block LSD1 protein interactions, not through inhibiting LSD1 catalytic activity. Interestingly, our data suggests that SP-2577 does not alter the interaction between LSD1 and CoREST1, which could be followed up on to design a LSD1 inhibitor with more specificity. An alternative hypothesis for why SP-2577 was the only clinical LSD1 inhibitor tested that blocked EEC differentiation is that SP-2577 has LSD1-independent effects. However, we find this hypothesis to be unlikely based on our similar results with LSD1 KD and the LSD1/CoREST inhibitor, corin. Another open question based on this study is how does BRAFi + EGFRi induce the interaction of LSD1-CoREST2-STAT3 to promote EEC differentiation. In previous work, we demonstrated baseline EEC differentiation in mucinous CRC is dependent on calcium [13]. It is possible that calcium signaling is also connected to the BRAFi + EGFRi induced increase in EECs as calcium signaling has been connected to BRAFi resistance in melanoma [35]. However, additional work is needed to explore this possibility.

Matched pretreatment and on treatment samples from patients with *BRAF*^*V600E*^ CRC are difficult to obtain because the cancers are rarely biopsied during treatment. In this study, we used the only publicly available dataset of paired pre and on-treatment samples from this patient group [32]. Similar to our preclinical models, on treatment samples had enrichment of secretory cell types, including EECs, compared to pretreatment samples. However, this analysis had several limitations. The sample size was small because only three of the 23 samples with pre and on treatment data had enough cells in the scRNAseq data to proceed with our analysis. Only two of the three samples contained any level of expression of EEC marker genes, suggesting that EEC enrichment occurs in a subset a patients with *BRAF*^*V600E*^ CRC receiving targeted therapy. Additionally, the patients received BRAFi (dabrafenib), MEKi (trametinib) and immunotherapy (anti-PD-1, sparatlizumab) because established dosing and safety data already existed for this regimen from patients with melanoma [32]. Whether or not the standard of care treatment for refractory metastatic *BRAF*^*V600E*^ CRC that consists of treatment with encorafenib (BRAFi) and cetuximab (EGFRi) has the same effect on cell type proportions needs to still be tested [2]. Interestingly, MEKi increased expression of marker genes for EECs and goblet cells in *BRAF*^*V600E*^ CRC cell lines, which is consistent with the on-treatment samples from the patient data having enrichment of several secretory cell types, not just EECs. Given the additional effects of BRAFi + MEKi + anti-PD-1 on lineage plasticity, teasing out the relative contributions of the different inhibitors and the interdependence of these effect on tumor growth will be challenging.

EECs in the normal colon secrete factors that regulate other epithelial and non-epithelial cells such as immune cells and neurons [8]. How EECs enriched in *BRAF*^*V600E*^ CRC following targeted therapy influence the tumor microenvironment will be explored in future studies using our newly developed syngeneic model of *BRAF*^*V600E*^ CRC. The stromal differences observed in tumors from this model in BRAFi + EGFRi versus vehicle treated mice suggest that treatment directly or indirectly through changes in tumor cell type influences stromal content. LSD1 inhibitors have also been used to increase tumor antigen presentation [36] so future work will also explore the effect of LSD1 inhibition on the immune response following BRAFi + EGFRi treatment of *BRAF*^*V600E*^ CRC.

Based on the body of work on the role of neuroendocrine transformation in therapy resistance in prostate and lung cancer and that EECs are neuroendocrine cells, we hypothesize that EEC enrichment following BRAFi + EGFRi in *BRAF*^*V600E*^ CRC also contributes to therapy resistance. Future work will explore the direct connection between EECs and therapy resistance in *BRAF*^*V600E*^ CRC by repeating in vivo experiments in models that have been experimentally depleted of EECs. Additional studies will also focus on determining whether the observed EEC enrichment is a transient state during treatment or if it is retained in resistant cells and/or tumors that start progressing on therapy. Furthermore, in vivo experiments using longer time points are needed to determine if LSD1 inhibition can prevent tumor progression and improve response to encorafenib plus cetuximab therapy.

Due to the results from the BREAKWATER clinical trial, encorafenib plus cetuximab treatment in combination with chemotherapy has now been FDA approved for the treatment of patients with treatment naïve microsatellite stable (MSS) *BRAF*^*V600E*^ CRC [3]. Additionally, the recommended treatment for patients with left-sided metastatic CRC with wildtype KRAS and BRAF is anti-EGFR antibody-based therapies, such as cetuximab, in combination with chemotherapy [37]. It is important to note that the NCI-H508 cells used in some of our experiments have a *BRAF*^*G596R*^ mutation, not the more common *BRAF*^*V600E*^ mutation, and patients with this mutation are not treated with encorafenib plus cetuximab [18]. Interestingly, MAPK inhibition in this cell line, mainly achieved by EGFRi, still enriches for EECs suggesting that MAPK inhibition but not *BRAF*^*V600E*^ is required for therapy-induced EEC enrichment. How BRAFi + EGFRi when used in combination with chemotherapy in *BRAF*^*V600E*^ CRC and EGFRi in BRAF/KRAS wildtype CRC alters cell plasticity and whether cell plasticity contributes to therapy resistance in these settings are important areas of future research. It will also be critical to determine if LSD1-CoREST2-STAT3 plays a role in cell plasticity in other subtypes of CRC or if it is restricted to *BRAF* mutant CRC treated with BRAFi + EGFRi as these findings will inform the group of CRC patients that have the potential to benefit from LSD1 inhibitor therapy.

## Conclusions

BRAFi + EGFRi treatment has limited effectiveness in patients with *BRAF*^*V600E*^ CRC [2]. Therapy-induced enrichment of EECs could contribute to resistance to this therapy regimen. Combining targeted therapy with epigenetic therapy such as an LSD1 inhibitor has the potential to improve patient responses to targeted therapy by blocking lineage plasticity.

## Electronic supplementary material

Below is the link to the electronic supplementary material.

Supplementary Material 1

Supplementary Material 2

Supplementary Material 3

Supplementary Material 4

Supplementary Material 5

Supplementary Material 6

## Data Availability

The scRNAseq datasets are accessible via the NCBI’s Gene Expression Omnibus (GEO) through GEO accession number GSE290028. Patient scRNAseq data analyzed in this study were obtained from https://singlecell.broadinstitute.org, study SCP2079. Only samples from patients that had greater than 200 cells for both pretreatment and on treatment samples were used in the analysis. All other raw data generated in this study are available upon request from the corresponding author.
