# Supplementary material for: LSD1 inhibition attenuates targeted therapy-induced lineage plasticity in BRAF mutant colorectal cancer
Source: Mol Cancer. 2025 Apr 23;24:122. doi: 10.1186/s12943-025-02311-z (PMC12016338; doi:10.1186/s12943-025-02311-z)
Supplement: Supplementary file 4 — Supplementary Material 4 [file 12943_2025_2311_MOESM4_ESM.pptx]

## Slide 1
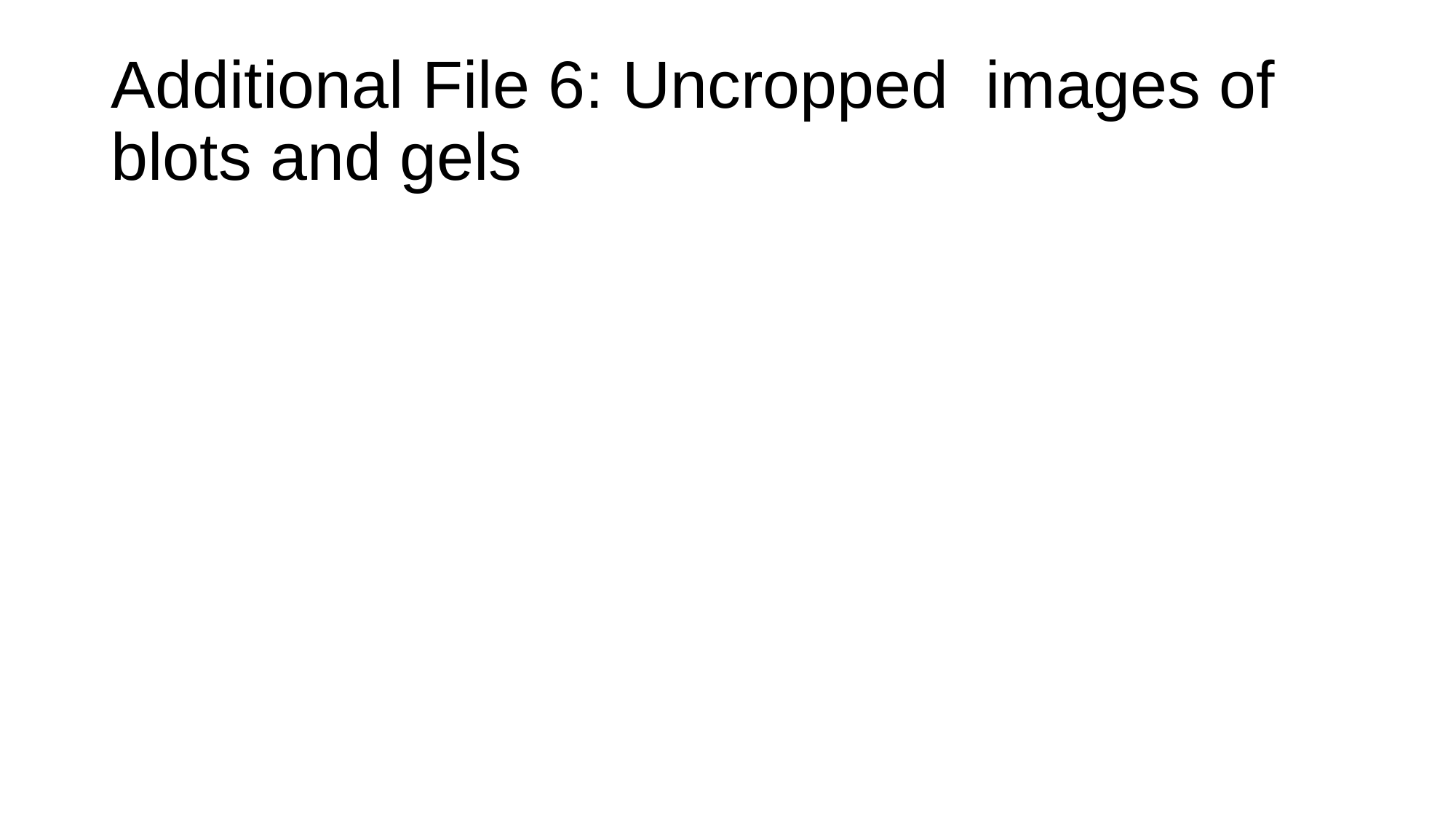

# Additional File 6: Uncropped images of blots and gels

## Slide 2
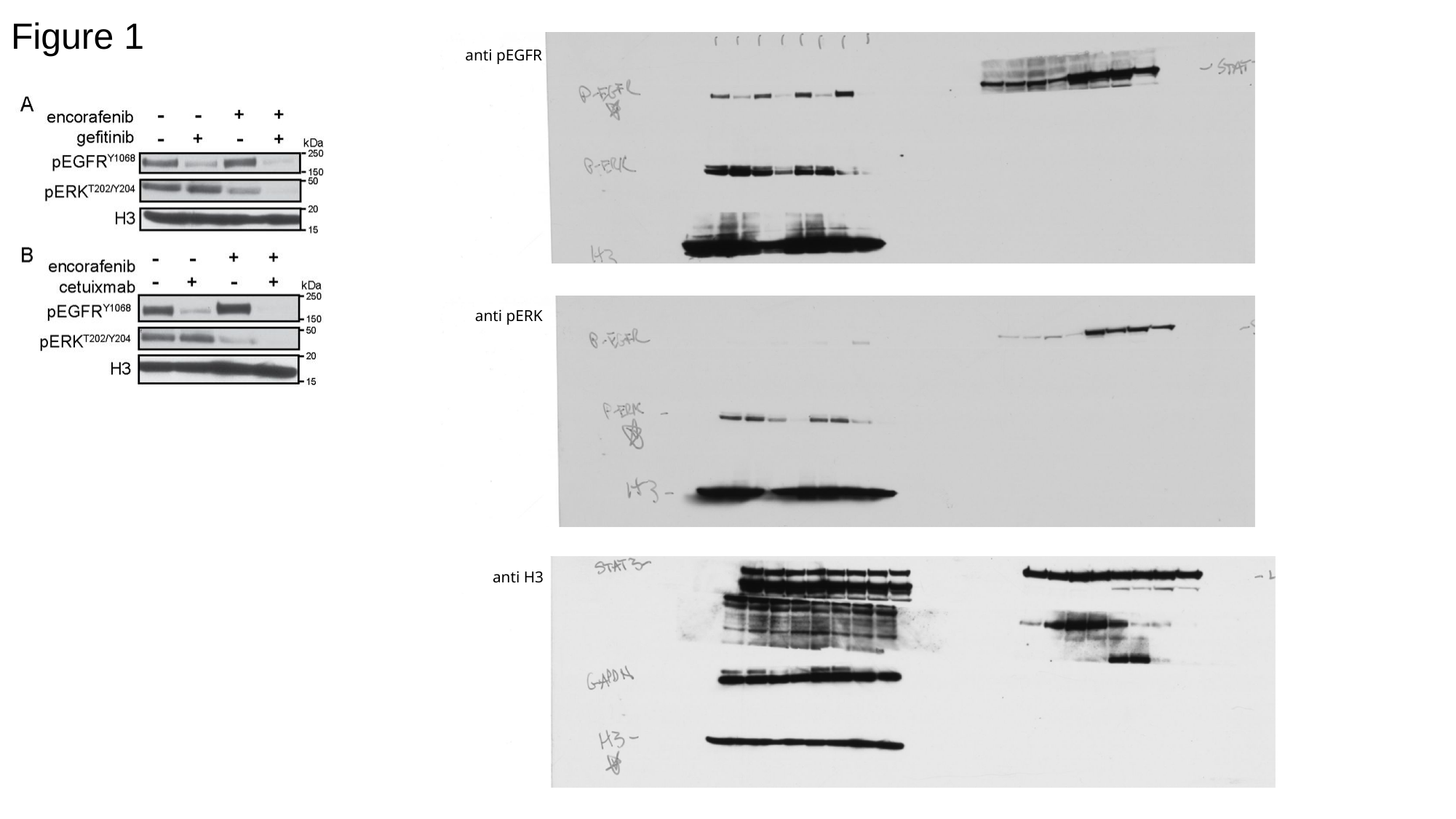

# Figure 1
anti pEGFR
anti pERK
anti H3

## Slide 3
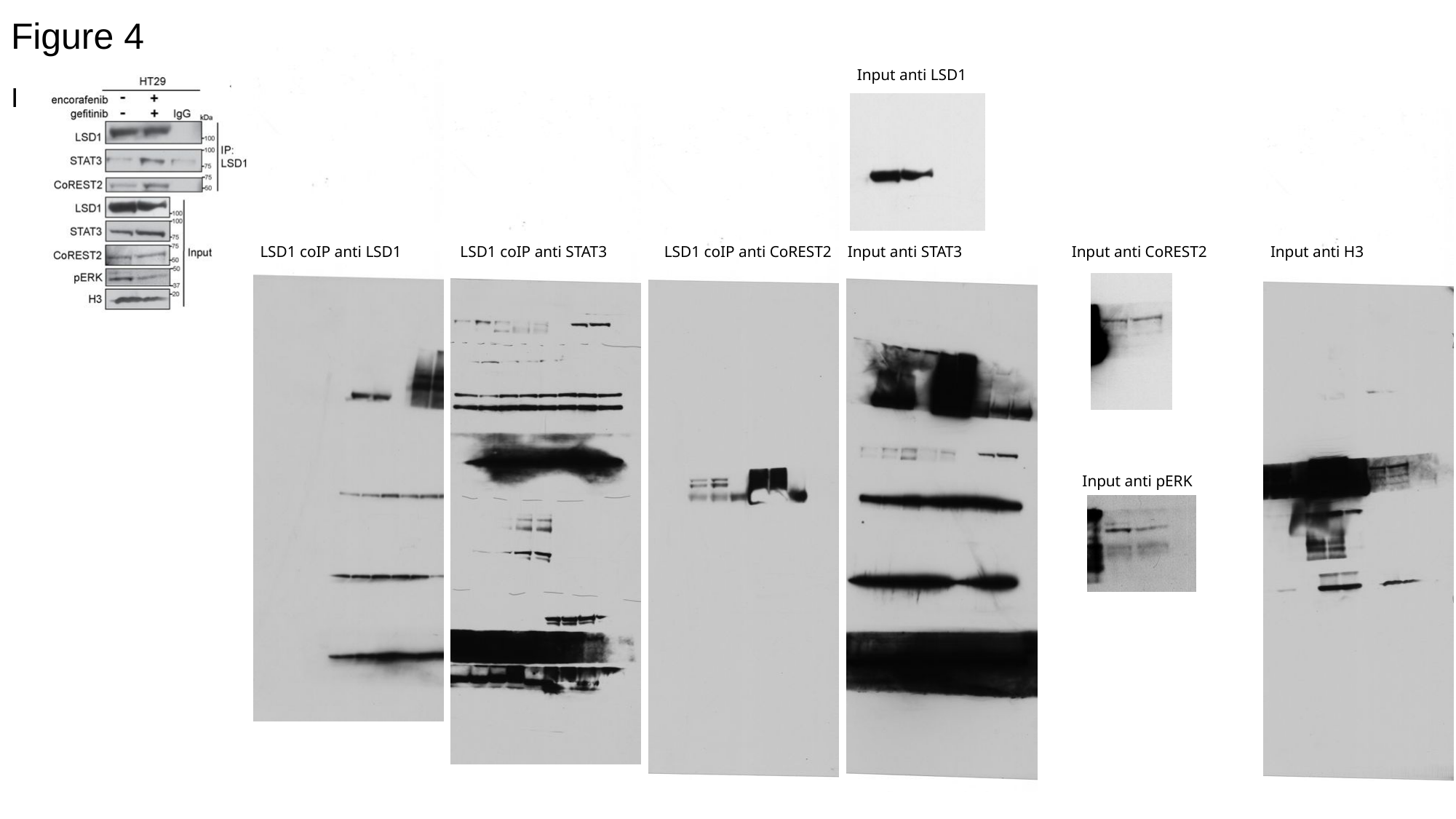

# Figure 4
Input anti LSD1
I
LSD1 coIP anti LSD1
LSD1 coIP anti STAT3
LSD1 coIP anti CoREST2
Input anti STAT3
Input anti H3
Input anti CoREST2
Input anti pERK

## Slide 4
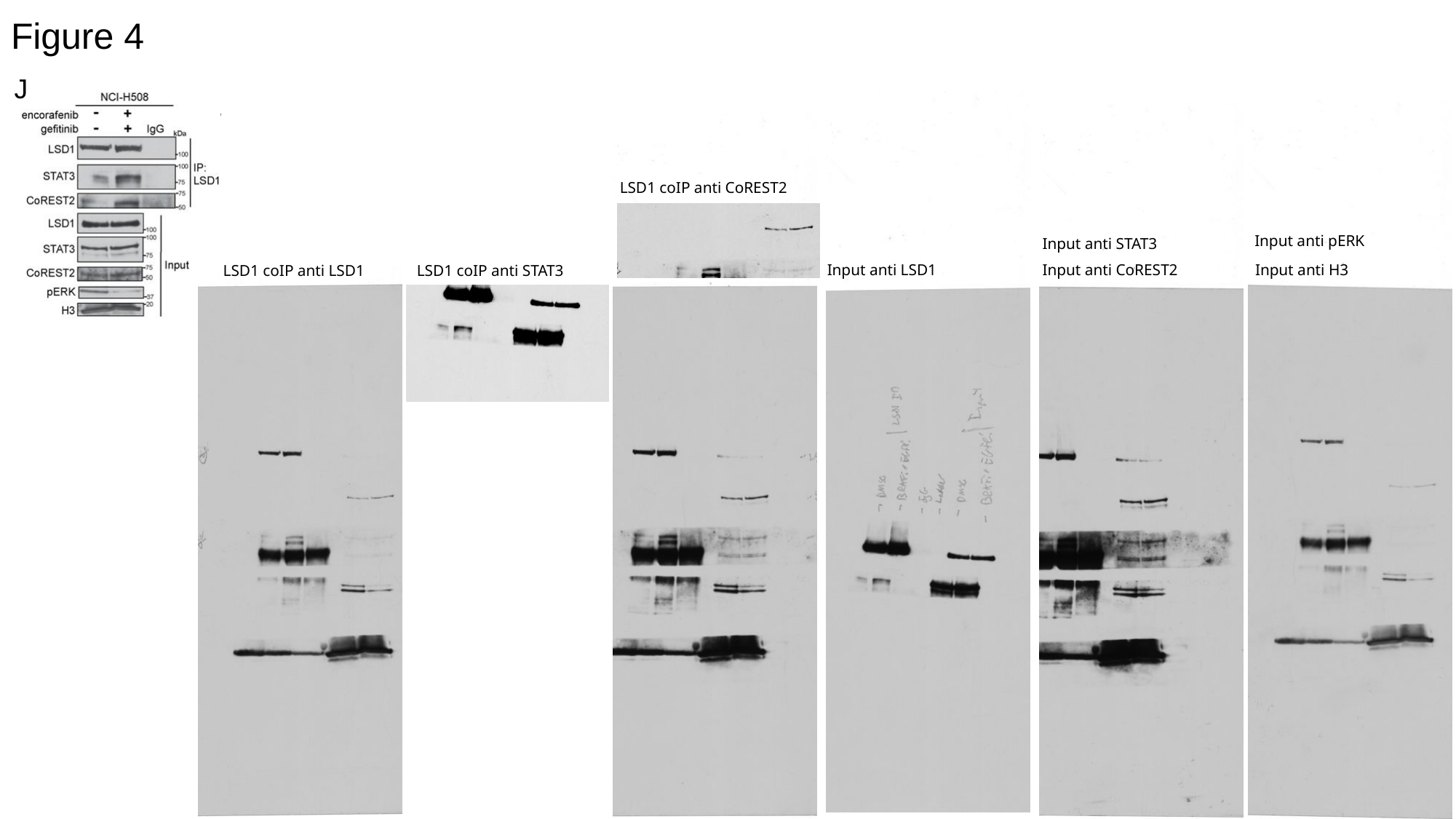

# Figure 4
J
LSD1 coIP anti CoREST2
Input anti pERK
Input anti STAT3
Input anti LSD1
Input anti CoREST2
Input anti H3
LSD1 coIP anti LSD1
LSD1 coIP anti STAT3

## Slide 5
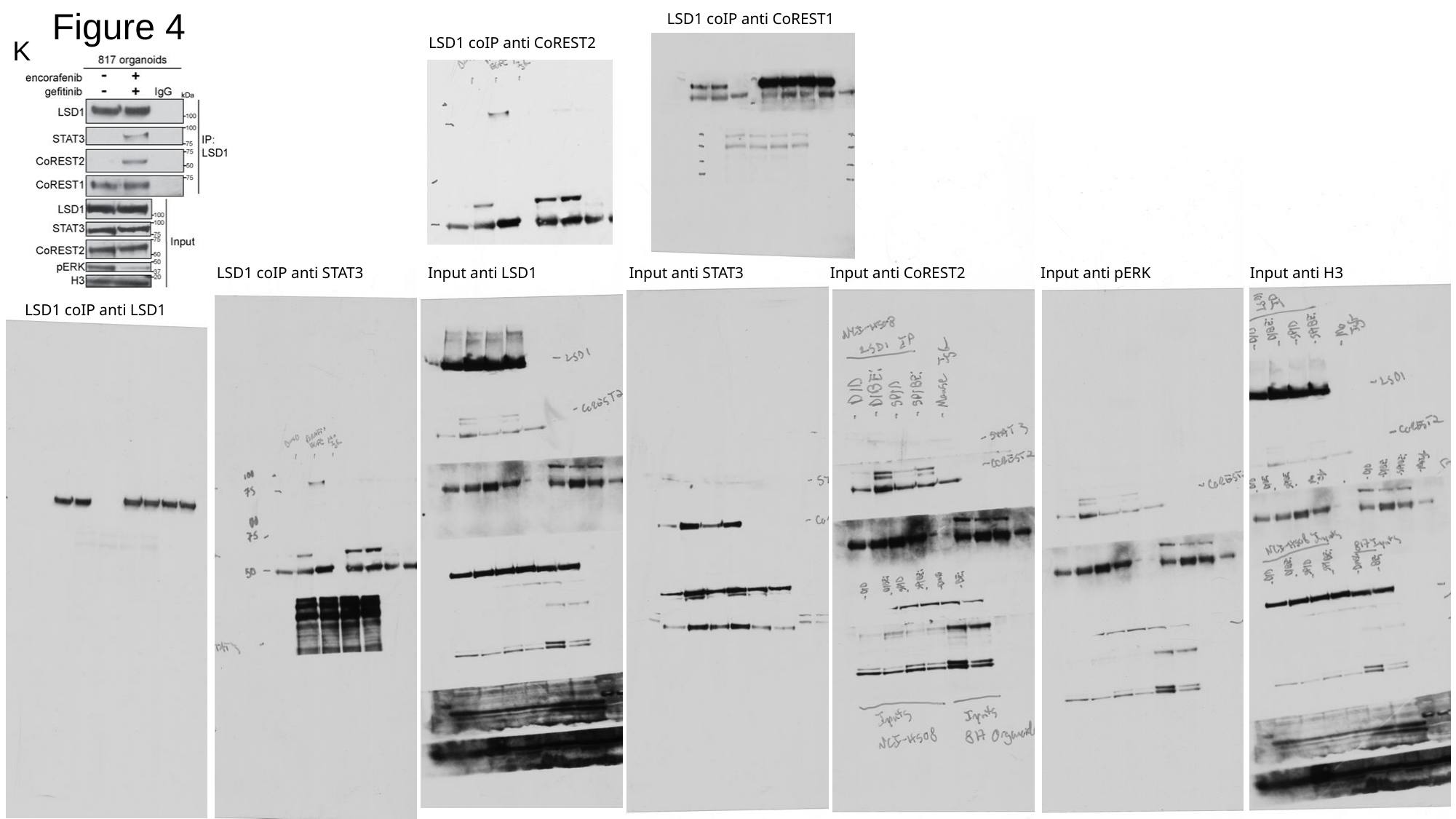

# Figure 4
LSD1 coIP anti CoREST1
K
LSD1 coIP anti CoREST2
LSD1 coIP anti STAT3
Input anti LSD1
Input anti STAT3
Input anti CoREST2
Input anti pERK
Input anti H3
LSD1 coIP anti LSD1

## Slide 6
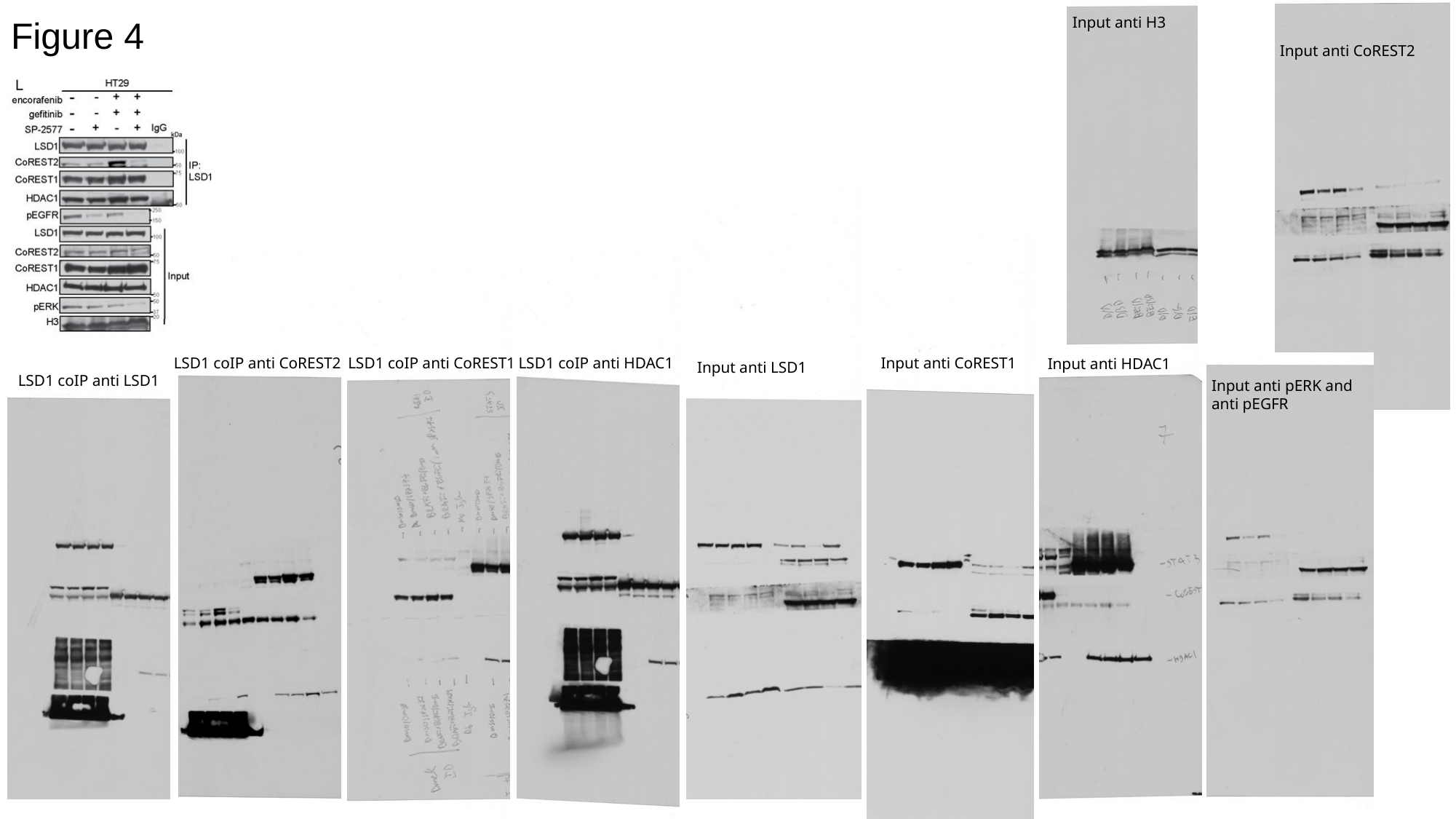

# Figure 4
Input anti H3
Input anti CoREST2
LSD1 coIP anti CoREST2
LSD1 coIP anti CoREST1
LSD1 coIP anti HDAC1
Input anti CoREST1
Input anti HDAC1
Input anti LSD1
LSD1 coIP anti LSD1
Input anti pERK and
anti pEGFR

## Slide 7
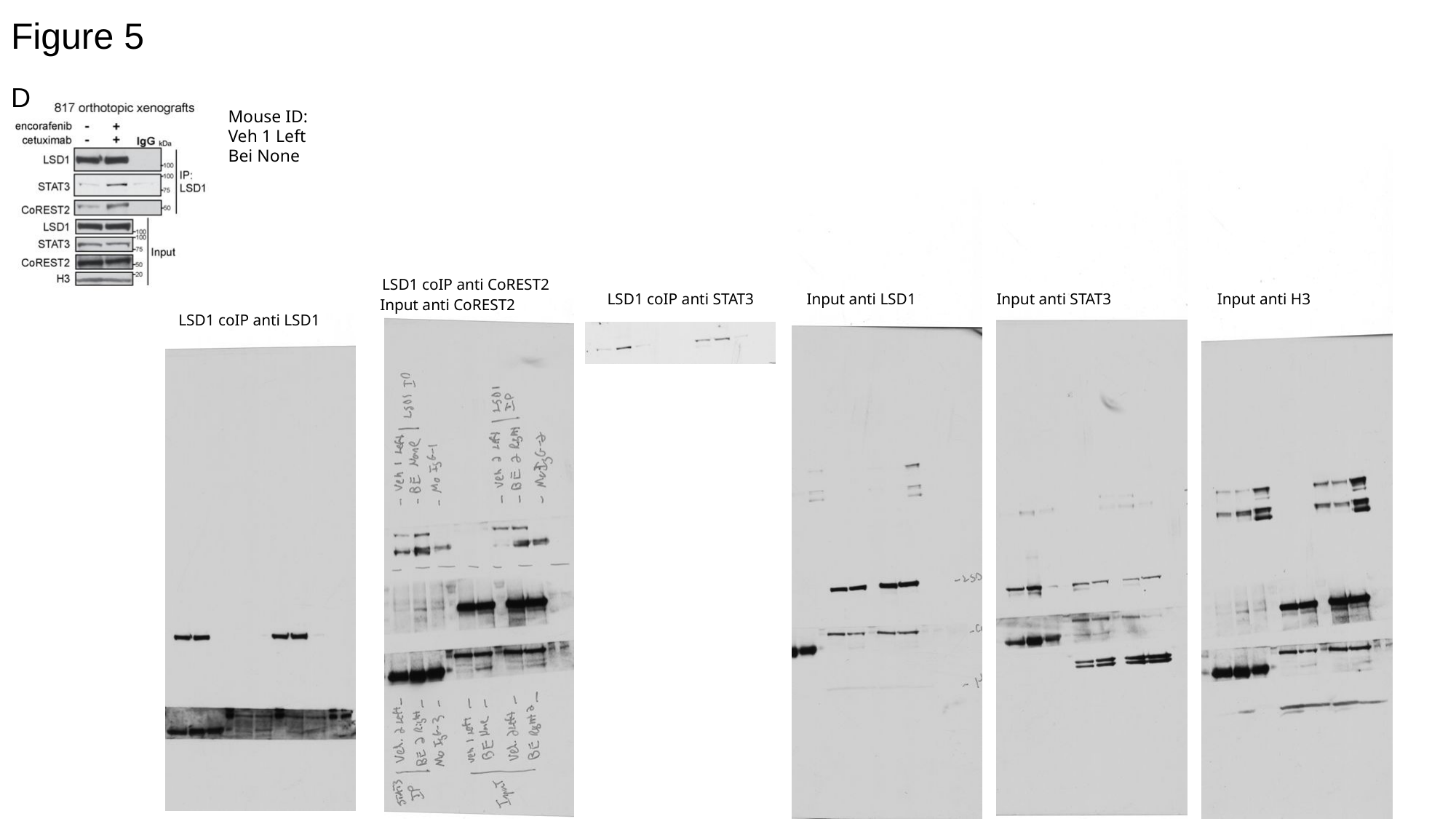

# Figure 5
D
Mouse ID:
Veh 1 Left
Bei None
LSD1 coIP anti CoREST2
Input anti H3
LSD1 coIP anti STAT3
Input anti LSD1
Input anti STAT3
Input anti CoREST2
LSD1 coIP anti LSD1

## Slide 8
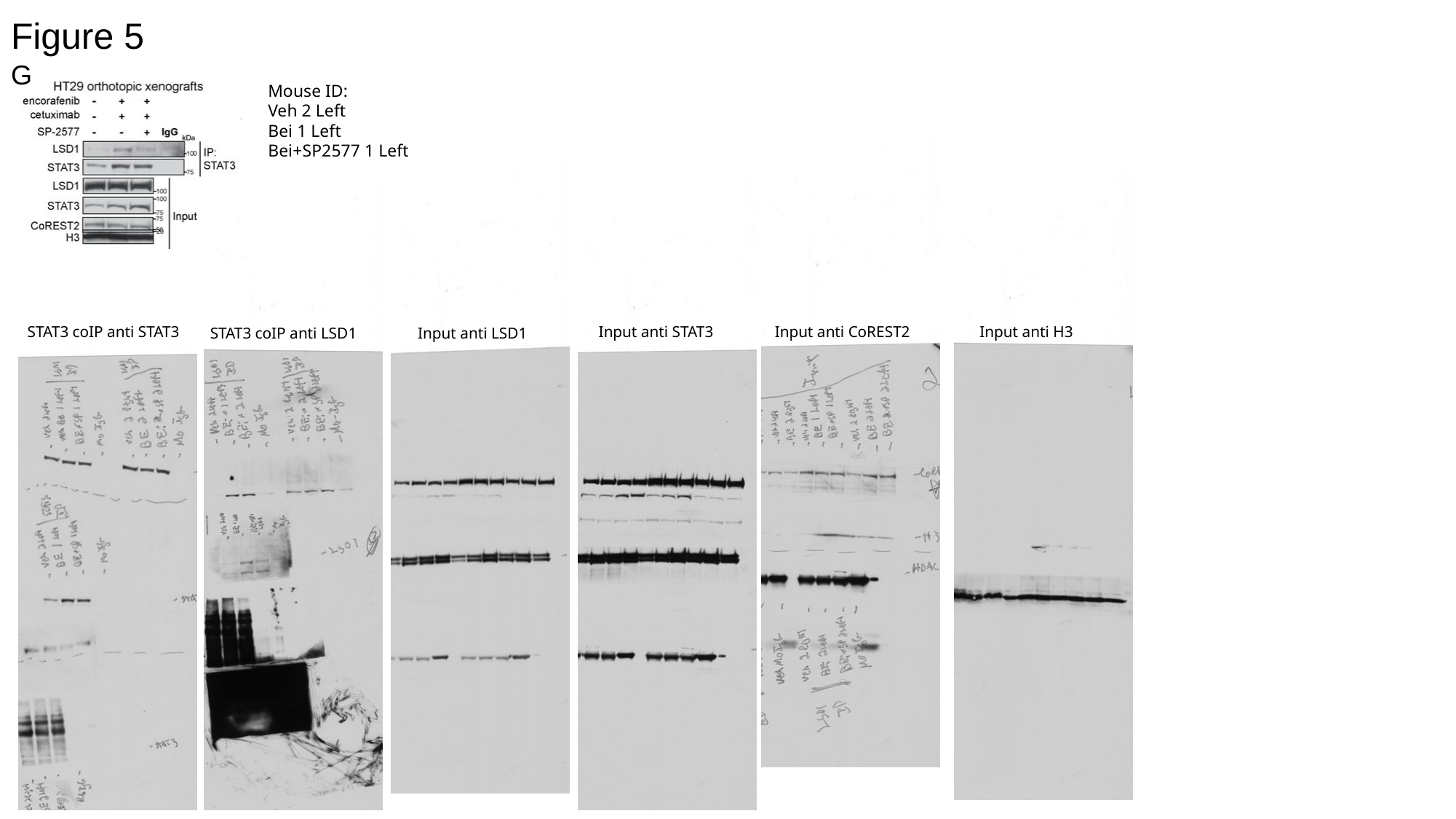

# Figure 5
G
Mouse ID:
Veh 2 Left
Bei 1 Left
Bei+SP2577 1 Left
STAT3 coIP anti STAT3
Input anti STAT3
Input anti CoREST2
Input anti H3
Input anti LSD1
STAT3 coIP anti LSD1

## Slide 9
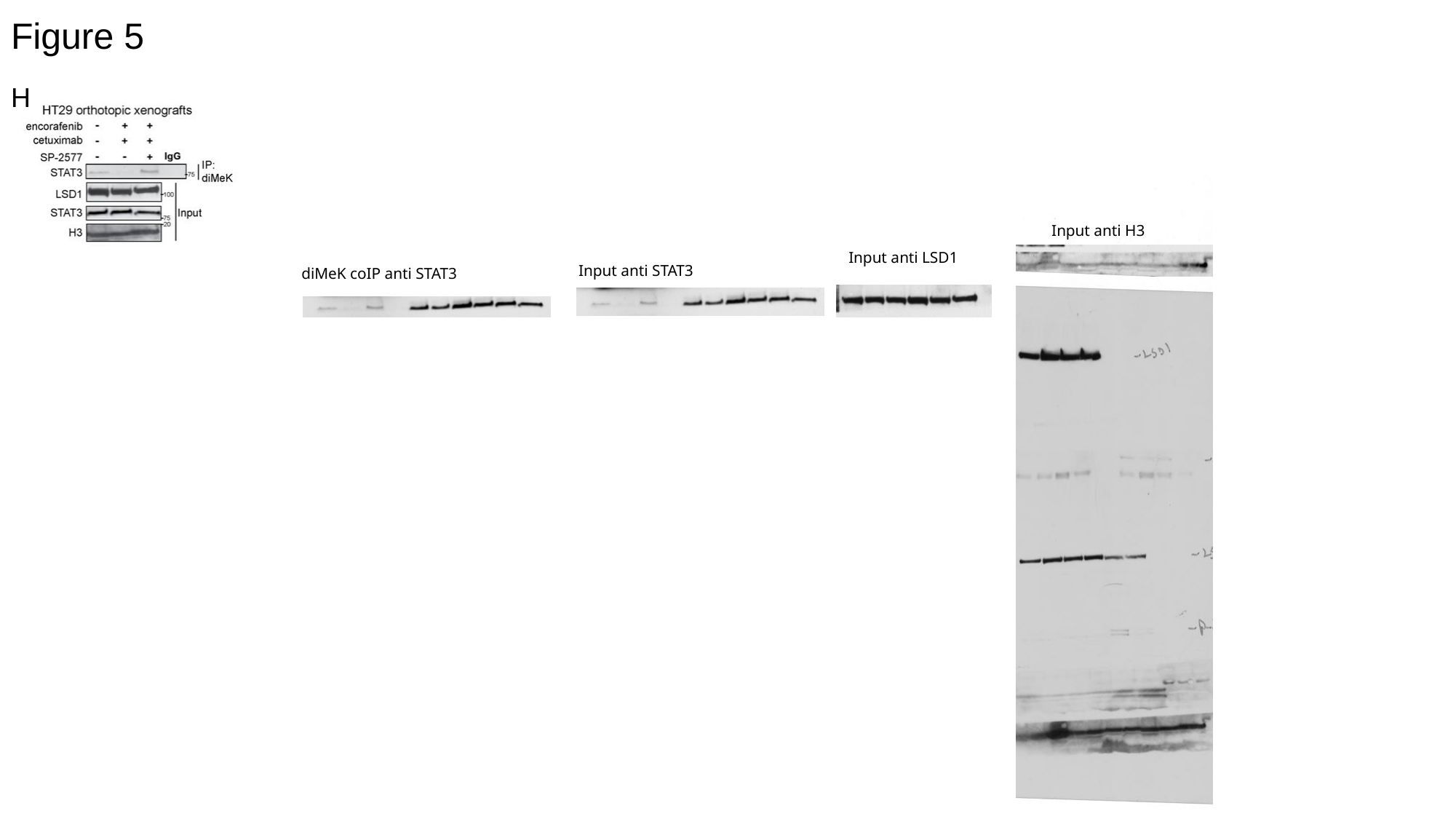

# Figure 5
H
Input anti H3
Input anti LSD1
Input anti STAT3
diMeK coIP anti STAT3

## Slide 10
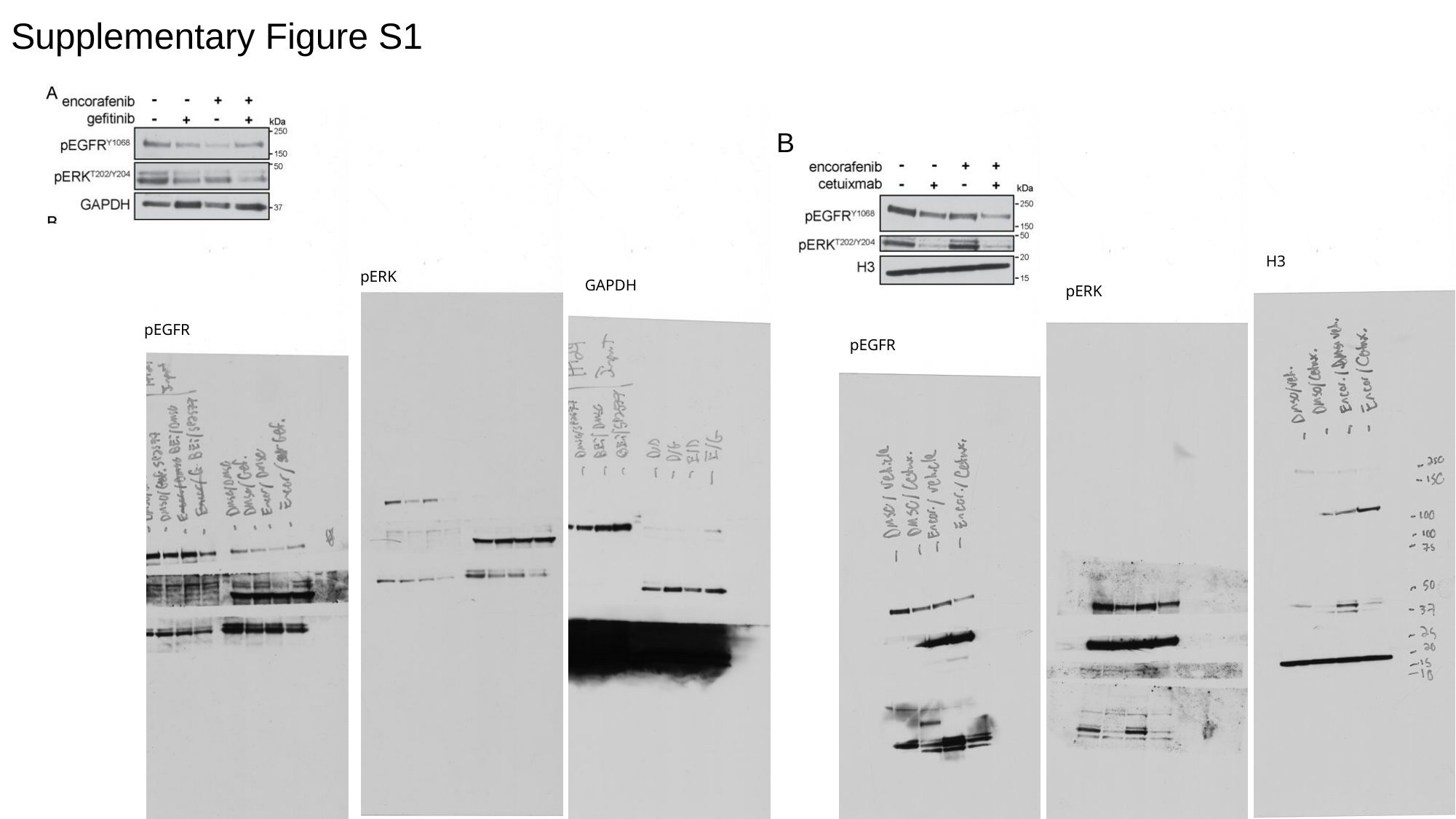

# Supplementary Figure S1
B
H3
pERK
GAPDH
pERK
pEGFR
pEGFR

## Slide 11
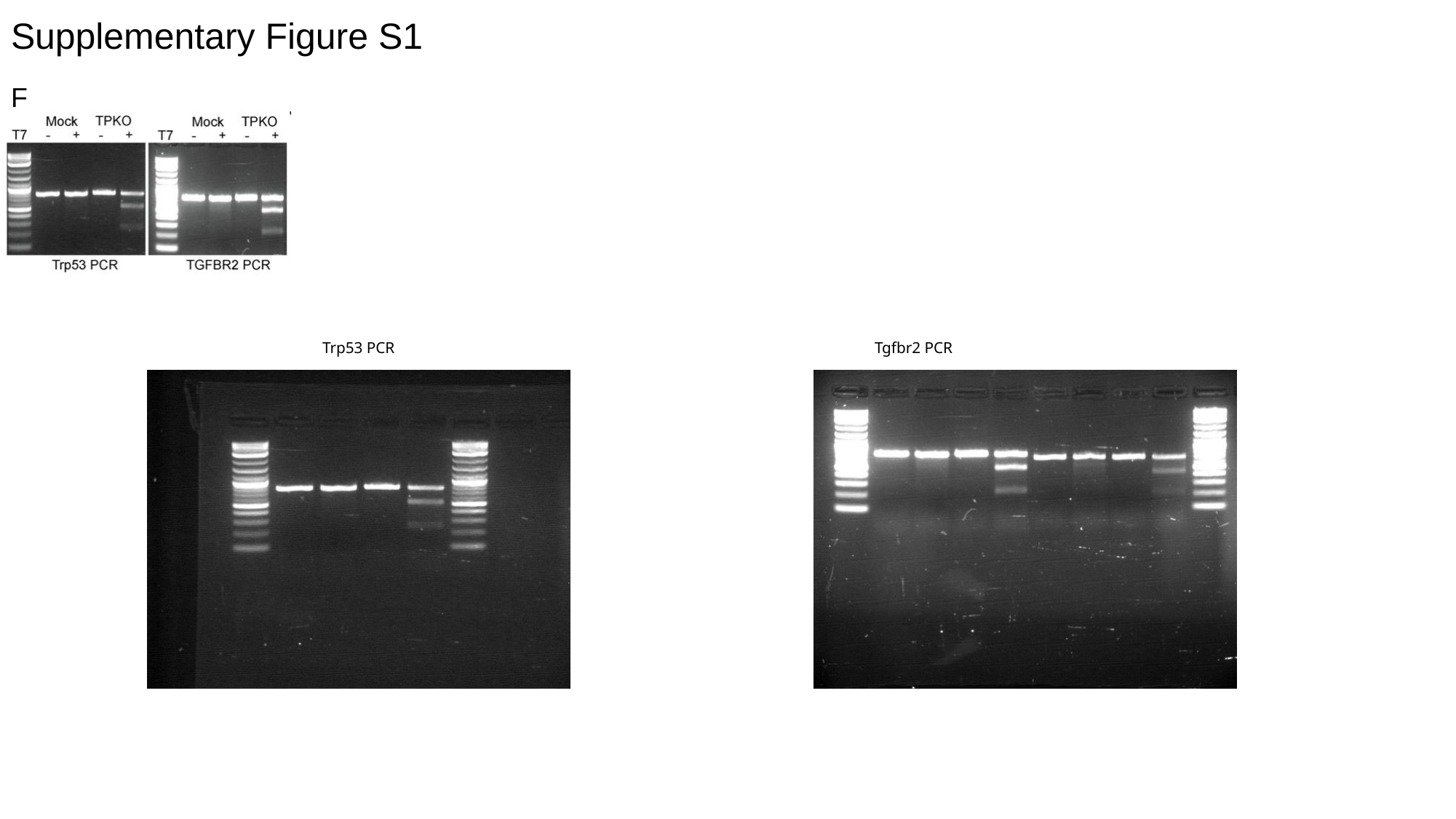

# Supplementary Figure S1
F
Trp53 PCR
Tgfbr2 PCR

## Slide 12
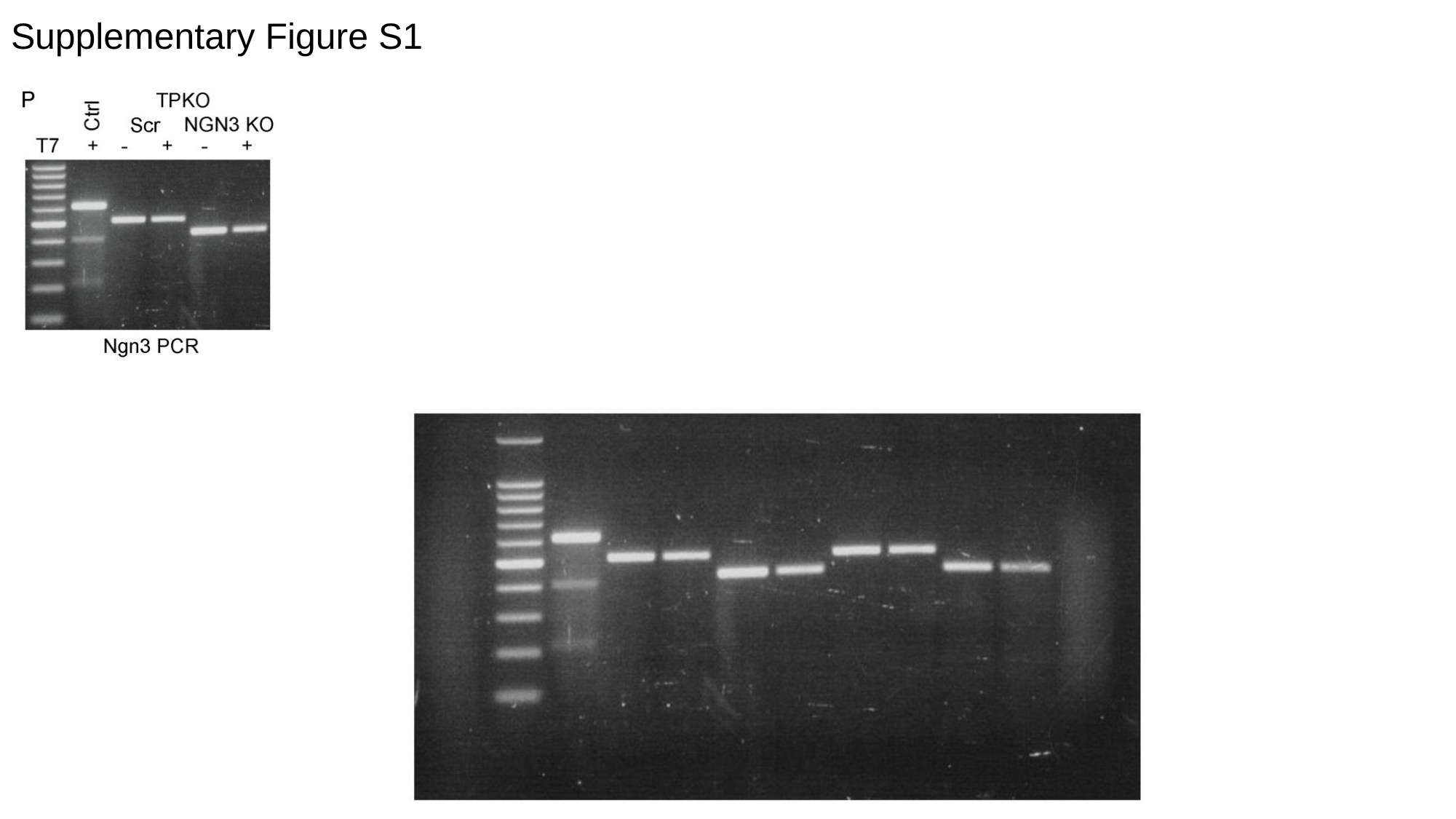

# Supplementary Figure S1

## Slide 13
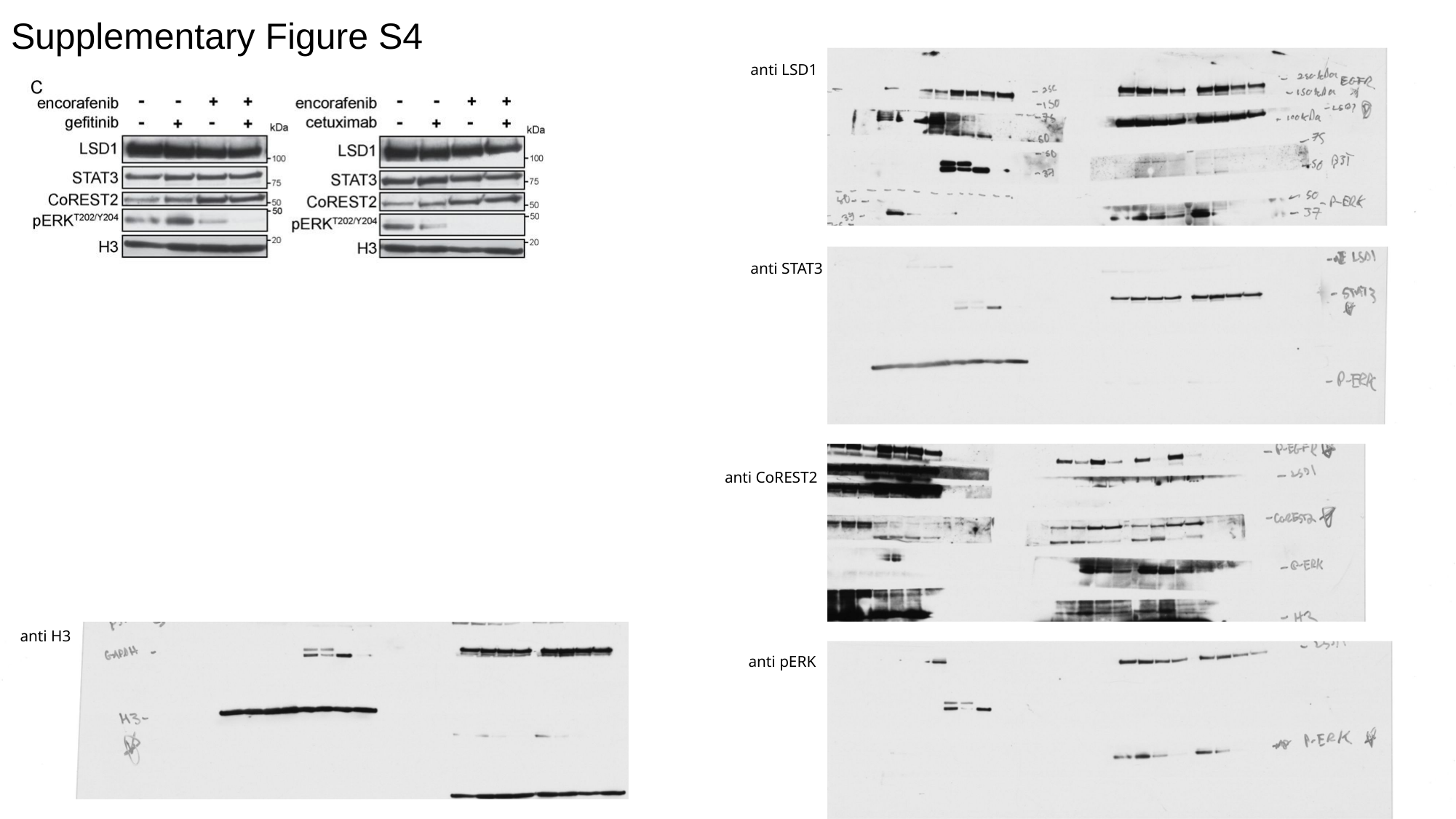

# Supplementary Figure S4
anti LSD1
anti STAT3
anti CoREST2
anti H3
anti pERK

## Slide 14
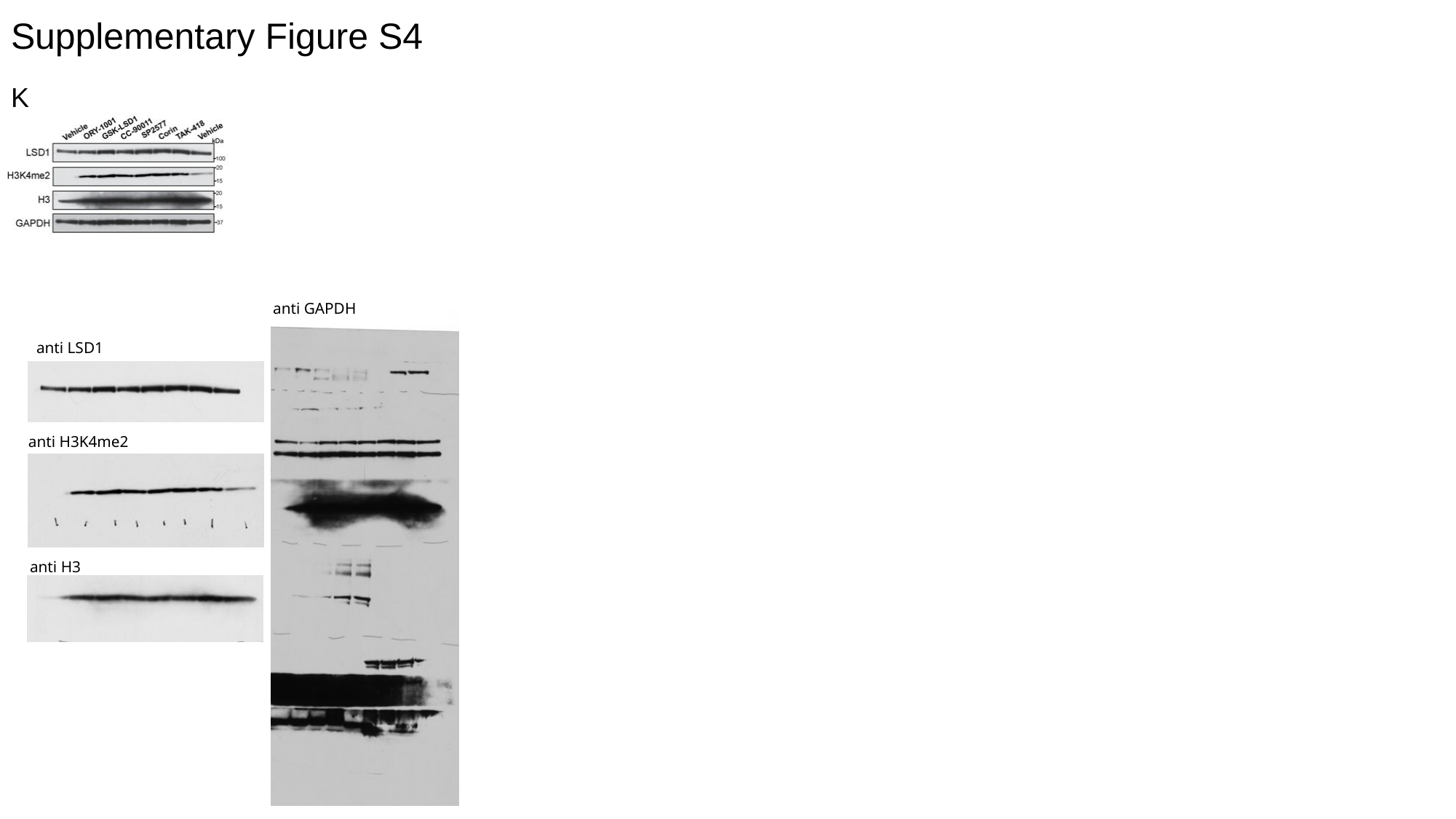

# Supplementary Figure S4
K
anti GAPDH
anti LSD1
anti H3K4me2
anti H3

## Slide 15
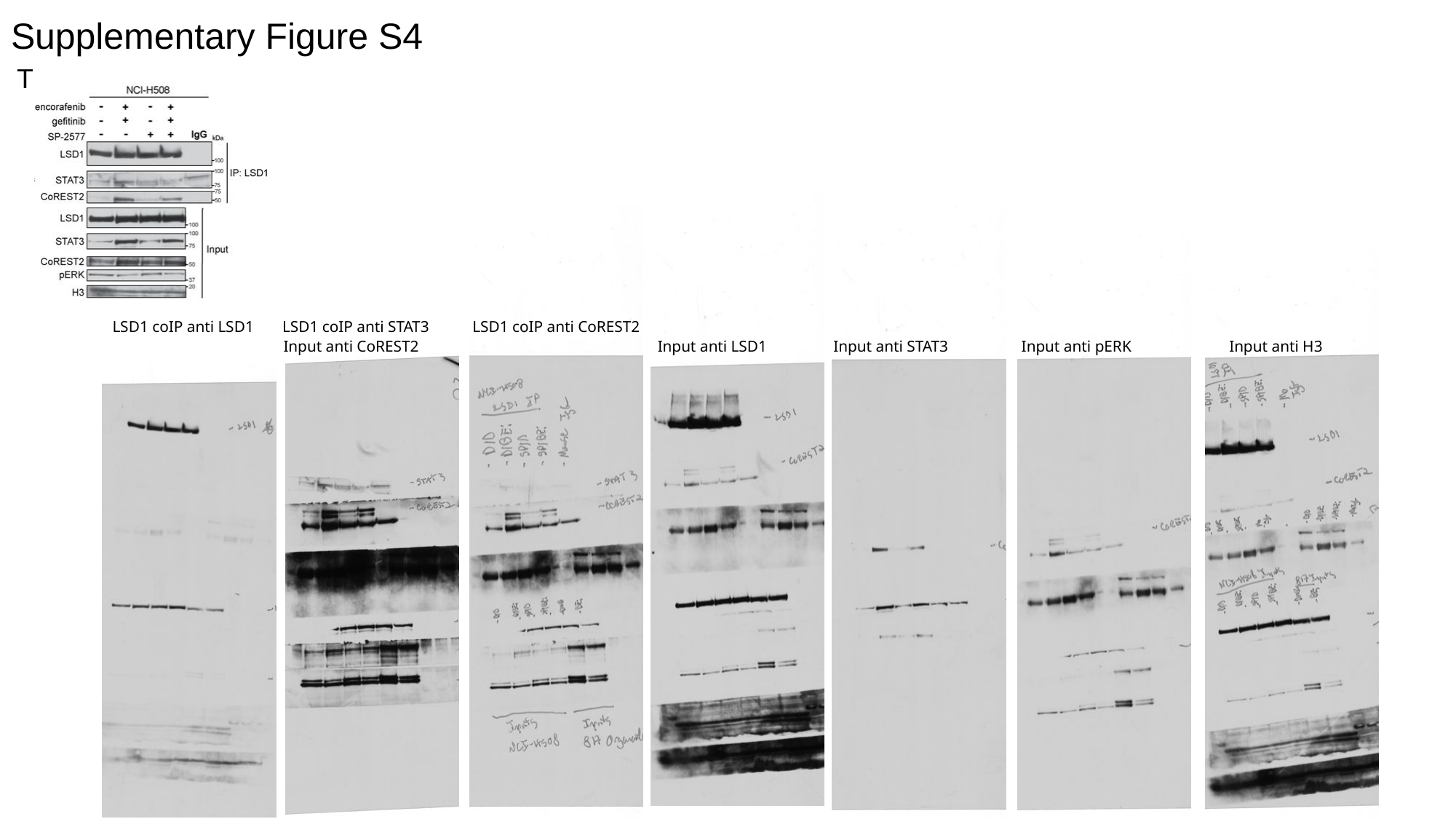

# Supplementary Figure S4
T
LSD1 coIP anti LSD1
LSD1 coIP anti STAT3
LSD1 coIP anti CoREST2
Input anti CoREST2
Input anti LSD1
Input anti STAT3
Input anti pERK
Input anti H3

## Slide 16
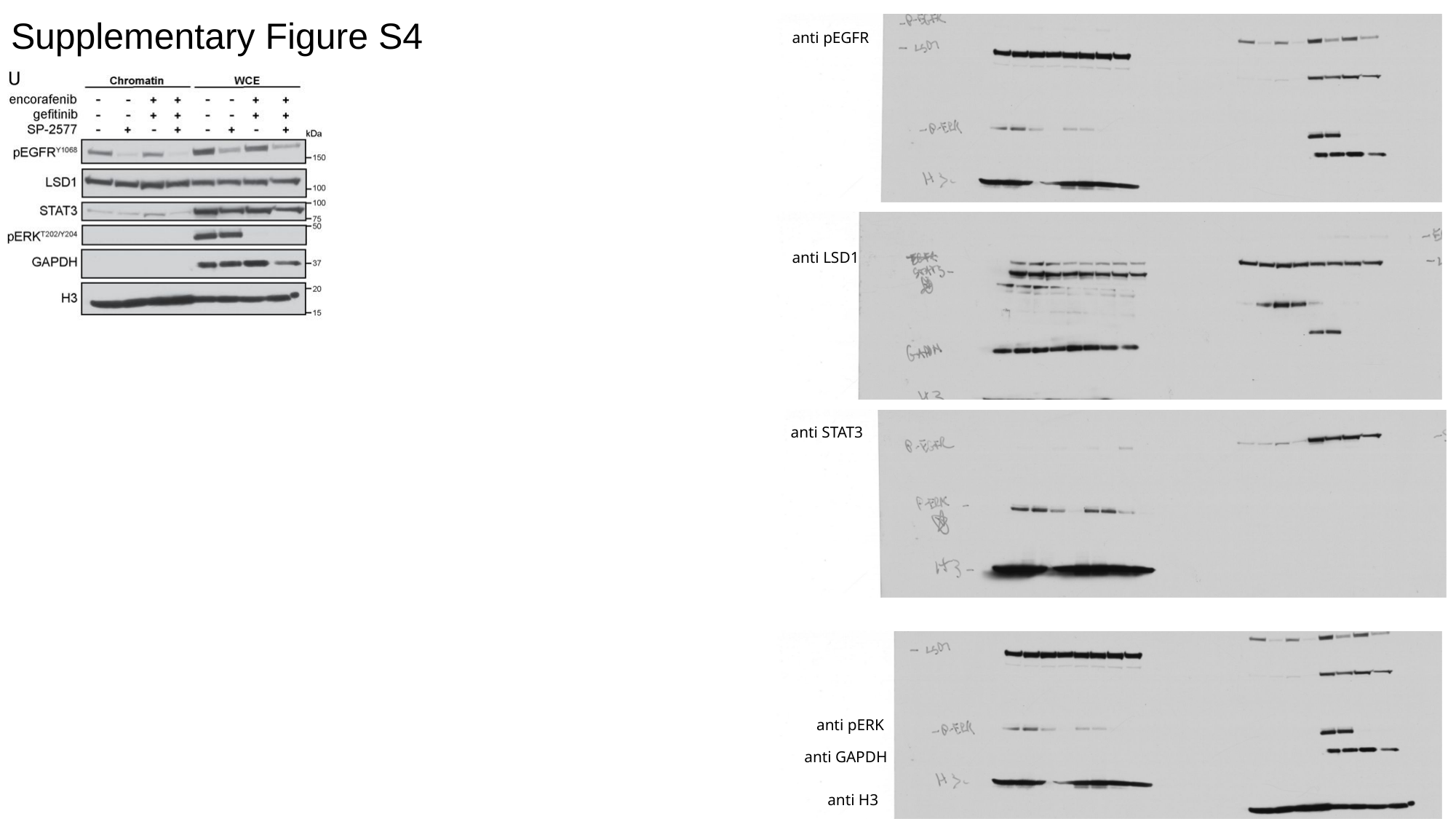

# Supplementary Figure S4
anti pEGFR
anti LSD1
anti STAT3
anti pERK
anti GAPDH
anti H3

## Slide 17
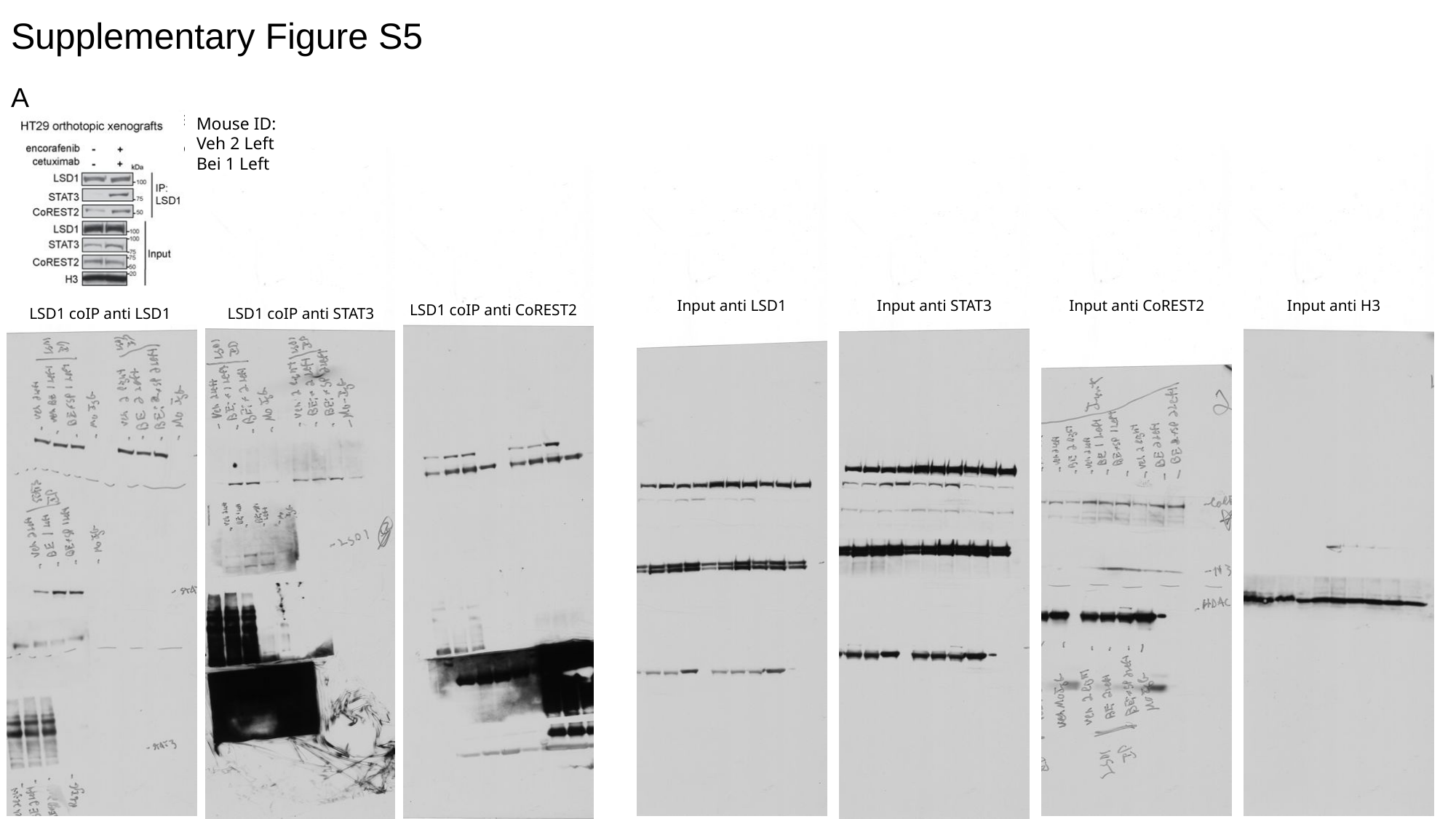

# Supplementary Figure S5
A
Mouse ID:
Veh 2 Left
Bei 1 Left
Input anti H3
Input anti STAT3
Input anti CoREST2
Input anti LSD1
LSD1 coIP anti CoREST2
LSD1 coIP anti LSD1
LSD1 coIP anti STAT3

## Slide 18
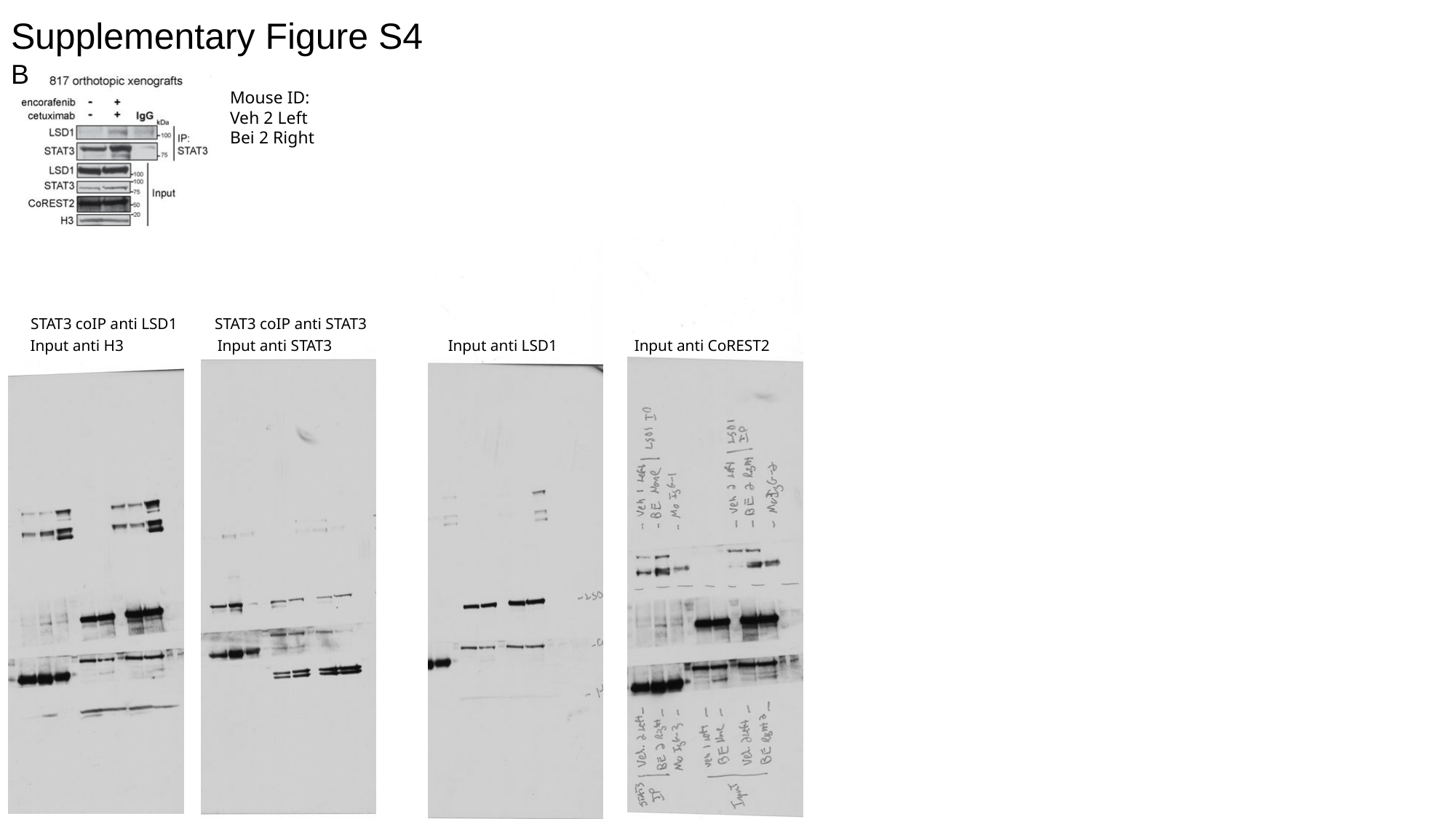

# Supplementary Figure S4
B
Mouse ID:
Veh 2 Left
Bei 2 Right
STAT3 coIP anti LSD1
STAT3 coIP anti STAT3
Input anti H3
Input anti STAT3
Input anti LSD1
Input anti CoREST2
