# Supplementary material for: LSD1 inhibition attenuates targeted therapy-induced lineage plasticity in BRAF mutant colorectal cancer
Source: Mol Cancer. 2025 Apr 23;24:122. doi: 10.1186/s12943-025-02311-z (PMC12016338; doi:10.1186/s12943-025-02311-z)
Supplement: Supplementary file 5 — Supplementary Material 5 [file 12943_2025_2311_MOESM5_ESM.docx]

**Supplementary Methods**

**Mouse colon CRC organoid derivation.**

C57BL/6J and *Braf^F-V600E^ Lgr5^tm1(Cre/ERT2)Cle^*Min*^ApcΔ716/+^* (BLM) mice were bred, handled, and inoculated with enterotoxigenic *Bacteroides fragilis* strain 86-5443-2-2 (ETBF) as in (1). Organoids were derived from proximal colon tumors 8 weeks after ETBF colonization as demonstrated previously and grown and passaged in basal growth media containing EGF (epidermal growth factor) and Noggin (2,3). TP KO organoids were generated by knocking out Trp53 and Tgfr2 in BLM tumor organoids. pRP[CRISPR]-Puro-hCas9-U6 plasmids were modified to contain gRNAs (mTrp53: ATGTGTAATAGCTCCTGCAT; mTgfr2: CACGAGGAGTACTCCTCGTA; VectorBuilder). The plasmids were cotransfected into dissociated organoids using lipofectamine 3000 and spinoculation. After replating in Matrigel, organoids were treated with 10 μM nutlin for 2 passages to select for knockout. Knockout was confirmed by T7 endonuclease assay using the EnGen Mutation Detection Kit (NEB #3321) and primers listed in supplementary Table S3. Scramble and Ngn3 KO TP KO organoids were generated as above using plasmids containing gRNAs Scramble: GTGTAGTTCGACCATTCGTG and mNeurog3: GCGATTGCGCTCCCGATCAT (VectorBuilder). Dissociated organoids were plated in 96 well plates and grown up to single organoids, which were screened for successful knockout by qRTPCR. The Ngn3 KO clone with the lowest Ngn3 expression was selected. A scramble clone with Ngn3 expression close to Ngn3 expression in the parental TP KO organoids was also selected.

**Tumor and tissue collection and analysis**

Even though the tumors are implanted in the colon, as they growth the tumor mass is externally visible and thus can be measured by calipers to determine tumor growth rates. Colon tumor size was measured twice weekly using calipers and volume was calculated as (length*width^2^)/2. Tissue from HT29 LUC2 tumors was assayed for luminescence ex-vivo by incubating for 5 minutes in 150 ug/ml luciferin and then imaging using an IVIS imaging system or plate reader. Colon tumors were fixed in formalin. Metastases in lungs from mice implanted with 817 organoids were assayed by inflating lungs with India ink dye and destaining in Fekete’s solution. White metastatic foci were counted. Following treatment, mice with TP KO orthoptic tumors were sacrificed and xenograft primary tumors, along with lymph nodes, lungs and livers were harvested.

**LSD1 knockdown**

LSD1 (TRCN0000046071 (KD1), TRCN0000327856 (KD2), TRCN0000327932 (KD3)) and empty vector TRC2 (#SHC201) knockdown constructs were purchased from Sigma Aldrich mission shRNA. Lentivirus were generated using the lentiviral shRNA knockdown protocol from The RNAi Consortium Broad Institute. To perform the transduction, virus-containing media and polybrene were added to cells. The next day, to select for transduced cells, cells were treated with puromycin (2µg/mL) (Sigma-Aldrich #P8833).

**Clonogenic growth assay**

1,500 HT29 cells were plated in a 6-well plate in McCoy’s +10% FBS for 1 week. After 1 week cells were treated as indicated every 48hrs for 6 days total (3x48hrs). Cells were then fixed with ice-cold methanol for 10 minutes and stained with crystal violet. Results were analyzed using ImageJ.

**Additional chemicals**

ORY-1001 (MedChemExpress #HY-109117), CC-90011 (MedChemExpress #HY-129388), T-3775440 (MedChemExpress #HY-103085), GSK-LSD1 (MedChemExpress #HY-100546), Corin (MedChemExpress #HY-111048), Binimetinib (MedChemExpress #HY-15202) and Stattic (MedChemExpress #HY-13818) were solubilized in DMSO to make 10 μM stock solutions and stored at -80^o^C.

**TCGA analysis**

TGFBR2 expression in the GDC TCGA Colon Cancer (COAD) was extracted using the Xena platform (4). Expression data was separated as normal colon samples, colon cancer samples with mutant *BRAF*, and colon cancer samples with wildtype *BRAF*.

**Details for Chromium Single Cell Flex Gene Expression**

**Sample preparation**

Briefly, minced tissue was fixed using 4% formaldehyde and *Concentrated Fix and Permeabilization Buffer* diluted in nuclease free water for 22 hours. After washing, tissue was resuspended in warm RPMI containing 0.2 mg/mL liberase and dissociated using the gentleMACS Dissociator (Miltenyi Biotec, 130-093-235), including incubation at 37^o^C for 30 minutes. Samples were filtered using through a 30 µm filter, and the dissociation reaction was quenched using *Concentrated Quench Buffer* (PN 2000516, 10X Genomics) diluted in nuclease free water. Cells were then resuspended in a 50% glycerol solution for storage at -80^o^C prior to sequencing.

**Pre-Processing and QC**

Filtered matrices were used to make a list of Seurat objects using the *Seurat* package v5.1.0 (5). Samples were normalized and filtered to only include cells with less than 5% mitochondrial content. Doublets were then removed using *DoubletFinder v2.0.4 (6)*. Variable features from normalized data were then identified using *FindVariableFeatures* and scaled using *ScaleData*. Dimension reduction was then performed using *RunPCA*. Integration of the list of Seurat objects was then performed using *IntegrateLayers* and join using the *JoinLayers* function. After integration the data was renormalized, scaled, and PCA reduction was performed.

**Visualizing scRNAseq data**

Module scores of genesets were calculated using the *AddModuleScore* function from the *Seurat* package. The data present in the Hallmarks heatmap was generated by applying the *AddModuleScore* to every Hallmark gene set present in the *misgdbr* package v7.5.1 and plotting the average module score for each cluster (7). Trajectory analysis was performed using the *monocle3* package v1.3.7 and manually annotating the stem cell cluster as the root cell (8).

| **Gene name** | **Forward primer** | **Reverse primer** |
| --- | --- | --- |
| mTrp53 T7 | ATAGAGACGCTGAGTCCGGT | GCCTGCGTACCTCTCTTTGC |
| mTgfbr2 T7 | AGTATCCGCGGACCCAGTTTA | GGTCACTGTGGAGATGAGCG |
| mNgn3 T7 | CCCGTGCAGTGACCTCTAAG | AGAAGCTGTGGTCCGCTATG |
| NGN3 | CTCACCAAGATCGAGACGCT | GTACAAGCTGTGGTCCGCTA |
| INSM1 | ACATCAACAAGTGCCACCCA | CGCACTCTCTTTGTGGGTCT |
| MUC2 | GCTATGTCGAGGACACCCAC | AGACGACTTGGGAGGAGTTG |
| HES1 | AAAAATTCCTCGTCCCCGGT | GGCTTTGATGACTTTCTGTGCT |
| LGR5 | AGACACGTACCCACAGAAGC | AACGCATTGTCATCCAGCCA |
| LSD1 (KDM1A) | GGTGAGCTCTTCCTCTTCTGG | TCGGCCAACAATCACATCGT |
| RCOR2 | TCAGCTCATCTCCCTCAAGC | CAGCGGGAGTTGAACTTGGT |
| RHOA | CGTTAGTCCACGGTCTGGTC | ACCAGTTTCTTCCGGATGGC |
| mTrp53 | GGAAGACTCCAGTGGGAACC | CTTCTGTACGGCGGTCTCTC |
| mBax1 | TGCTGATGGCAACTTCAACTG | CTGATCAGCTCGGGCACTTTA |
| mTgfbr2 | GAGTCGTTCAAGCAGACGGA | GAACCAAATGGGGGCTCGTA |
| mRb1 | ATGGAATCCCTTGCATGGCT | GCTGAGAGGACAAGCAGGTT |
| mNgn3 | GTTCCAATTCCACCCCACCT | GTTTGCTGAGTGCCAACTCG |
| mSyp | TGTGCCAACAAGACGGAGAG | TAGTGCCCCCTTTAACGCAG |
| mTubb3 | CCTATTCAGGCCCGACAACTT | CCTGCAGGCAGTCACAATTC |
| mChga | GCAAGTTTTTGCCCTTCCTGT | CTGGGTTTGGACAGCGAGT |
| **Taqman assays** | | |
| Neurog3 | Mm00437606_s1 |  |
| Insm1 | Mm02581025_s1 |  |
| Muc2 | Mm01276696_m1 |  |
| Hes1 | Mm01342805_m1 |  |
| Lgr5 | Mm00438890_m1 |  |
| Ppia | Mm02342430_g1 |  |

**Supplementary Table S4. Primer sequences and Taqman assays.**

| **Antibody** | **Company** | **Product #** | **Assay and dilution** |
| --- | --- | --- | --- |
| Anti-β3-tubulin | R&D Systems | MAB1195-SP | IF 1:500, IHC 1:000 (Citrate HIER) |
| Anti-phospho-ERK | CST | 4370 | IHC 1:100 (Citrate HIER), Western blot |
| Anti-Ki67 | CST | 9449 | IHC 1:1000 (Citrate HIER) |
| Anti-INSM1 | Sigma-Aldrich | 475R-98 | IHC (Tris/EDTA HIER) |
| Anti-Synaptophysin | CST | 36406 | IHC 1:200 (Citrate HIER) |
| Anti-PROX1 | Abcam | Ab199359 | IHC 1:500 (Tris/EDTA HIER) |
| Anti-LSD1 | CST | 4218 | CoIP |
| Anti-LSD1 | CST | 2139 | Western blot |
| Anti-STAT3 | CST | 9139 | Western blot, CoIP |
| Anti-CoREST2 | Sigma | HPA021638 | Western blot |
| Anti-H3 | CST | 9717 | Western blot |
| Anti-GAPDH | CST | 5714 | Western blot |
| Anti-CoREST1 | CST | 14567 | Western blot |
| Anti-HDAC1 | CST | 5356 | Western blot |
| Anti-H3K4me2 | Millipore | 17-677 | Western blot |
| Anti-phospho-EGFR | CST | 2234 | Western blot |
| Anti-EGFR | CST | 2232 | Western blot |
| Anti-Di-Methyl Lysine Motif | CST | 14117 | CoIP |

**Supplementary Table S5. Antibodies used.**

**Supplementary** **References**

1. DeStefano Shields CE, White JR, Chung L, Wenzel A, Hicks JL, Tam AJ*, et al.* Bacterial-driven inflammation and mutant BRAF expression combine to promote murine colon tumorigenesis that is sensitive to immune checkpoint therapy. Cancer discovery **2021**;11:1792-807.

2. Mahe MM, Aihara E, Schumacher MA, Zavros Y, Montrose MH, Helmrath MA*, et al.* Establishment of Gastrointestinal Epithelial Organoids. Current protocols in mouse biology **2013**;3:217-40.

3. Xue X, Shah YM. In vitro organoid culture of primary mouse colon tumors. Journal of visualized experiments : JoVE **2013**:e50210.

4. Goldman MJ, Craft B, Hastie M, Repečka K, McDade F, Kamath A*, et al.* Visualizing and interpreting cancer genomics data via the Xena platform. Nature Biotechnology **2020**;38:675-8.

5. Hao Y, Hao S, Andersen-Nissen E, Mauck WM, III, Zheng S, Butler A*, et al.* Integrated analysis of multimodal single-cell data. Cell **2021**;184:3573-87.e29.

6. McGinnis CS, Murrow LM, Gartner ZJ. DoubletFinder: Doublet Detection in Single-Cell RNA Sequencing Data Using Artificial Nearest Neighbors. Cell Systems **2019**;8:329-37.e4.

7. Dolgalev I. msigdbr: MSigDB gene sets for multiple organisms in a tidy data format. R Package Version **2024**

8. Cao J, Spielmann M, Qiu X, Huang X, Ibrahim DM, Hill AJ*, et al.* The single-cell transcriptional landscape of mammalian organogenesis. Nature **2019**;566:496-502.
