# Supplementary material for: LSD1 inhibition attenuates targeted therapy-induced lineage plasticity in BRAF mutant colorectal cancer
Source: Mol Cancer. 2025 Apr 23;24:122. doi: 10.1186/s12943-025-02311-z (PMC12016338; doi:10.1186/s12943-025-02311-z)
Supplement: Supplementary file 6 — Supplementary Material 6 [file 12943_2025_2311_MOESM6_ESM.pdf]

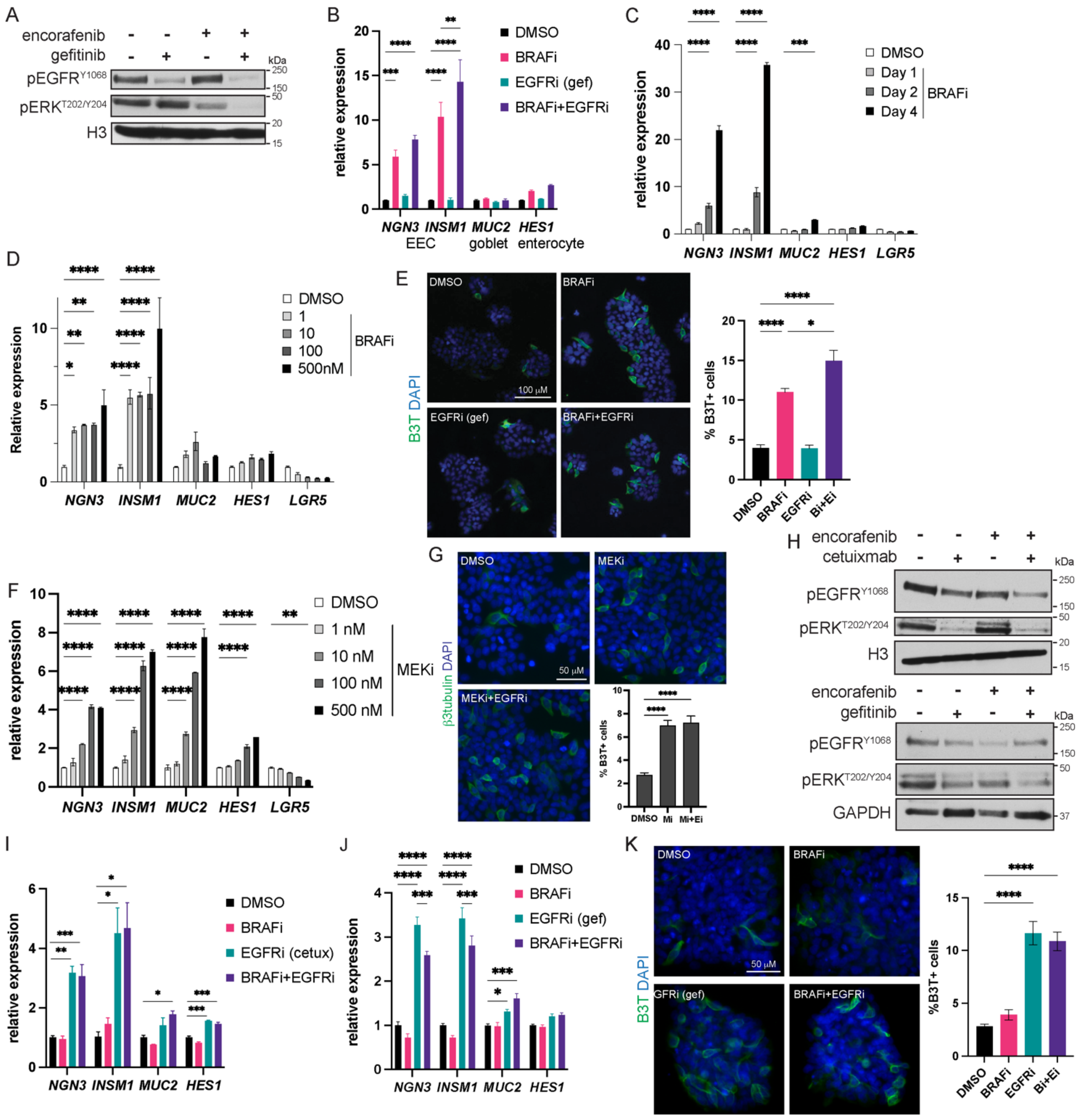
A
encorafenib
gefitinib
pEGFR Y1068
pERK T202/Y204
H3
kDa
B
relative expression
NGN3 INSM1 MUC2 HES1
EEC goblet enterocyte
DMSO
BRAFi
EGFRi (gef)
BRAFi+EGFRi
C
relative expression
NGN3 INSM1 MUC2 HES1 LGR5
DMSO
Day 1
Day 2
Day 4
BRAFi
D
Relative expression
NGN3 INSM1 MUC2 HES1 LGR5
DMSO
1
10
100
500nM
BRAFi
E
B3T DAPI
DMSO
BRAFi
EGFRi (gef)
BRAFi+EGFRi
100 μM
% B3T+ cells
DMSO BRAFi EGFRi Bi+Ei
F
relative expression
NGN3 INSM1 MUC2 HES1 LGR5
DMSO
1 nM
10 nM
100 nM
500 nM
MEKi
G
β3tubulin DAPI
DMSO
MEKi
MEKi+EGFRi
50 μM
% B3T+ cells
DMSO Mi Mi+Ei
H
encorafenib
cetuixmab
pEGFR Y1068
pERK T202/Y204
H3
encorafenib
gefitinib
pEGFR Y1068
pERK T202/Y204
GAPDH
kDa
I
relative expression
NGN3 INSM1 MUC2 HES1
DMSO
BRAFi
EGFRi (cetux)
BRAFi+EGFRi
J
relative expression
NGN3 INSM1 MUC2 HES1
DMSO
BRAFi
EGFRi (gef)
BRAFi+EGFRi
K
B3T DAPI
DMSO
BRAFi
GFRi (gef)
BRAFi+EGFRi
50 μM
%B3T+ cells
DMSO BRAFi EGFRi Bi+Ei

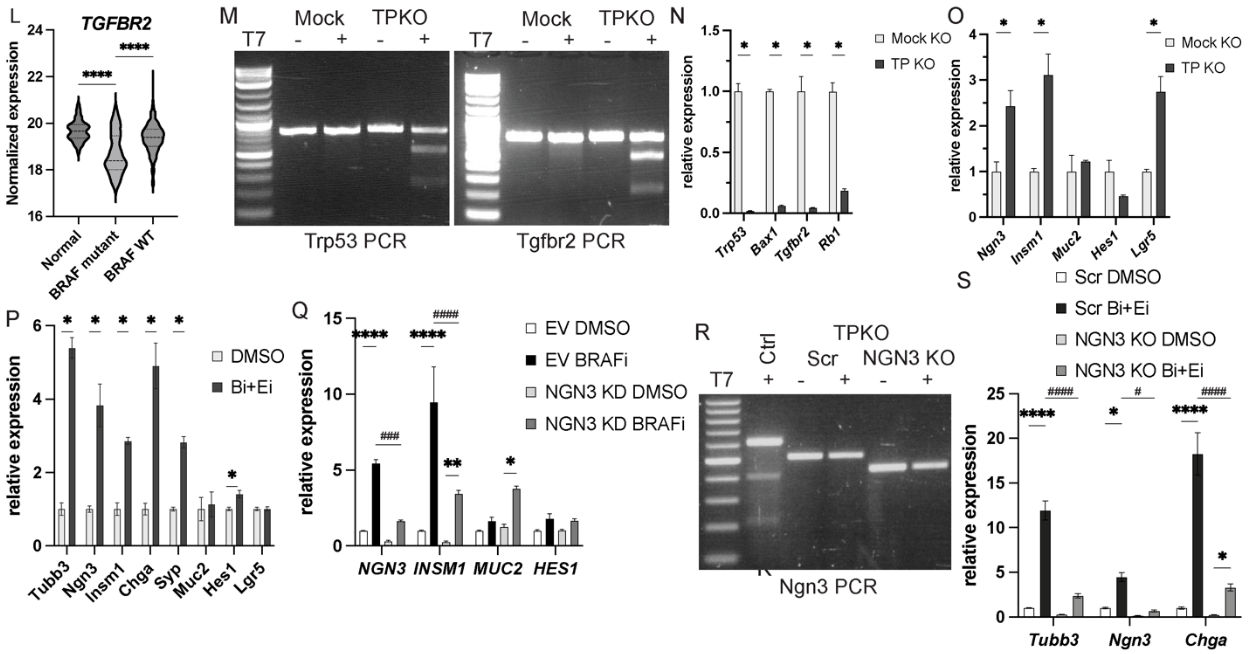

Supplementary Figure S1. MAPK pathway inhibition enriches for EECs in *BRAF* mutant CRC. A) Western blots of HT29 cells treated with DMSO or 2.5 nM encorafenib (BRAFi) alone or in combination with 500 nM gefitinib (EGFRi, gef) for 48H. (B) Relative gene expression of indicated genes in HT29 cells treated as in A. Gene expression was normalized to the housekeeping gene *RHOA* and then to the DMSO treated cells. Graph represents mean +/- SEM. N=3. (C) Relative gene expression of indicated genes in HT29 cells treated with DMSO or 2.5 nM encorafenib (BRAFi) for the indicated number of days. Data presented as in B. (D) Relative gene expression of the indicated genes in HT29 cells treated with the indicated concentration of encorafenib (BRAFi) for 48H. Data presented as in B. (E) Immunofluorescence for β3-tubulin (B3T) in HT29 cells treated as in A for 72H. Graph is the %β3-tubulin+ cells of the total number of cells per field. N=3. (F) Relative gene expression of indicated genes in HT29 cells treated with the indicated concentrations of binimetinib (MEKi) for 48H. Data presented as in B. (G) Immunofluorescence for β3-tubulin (B3T) in HT29 cells treated with 50 nM binimetinib (MEKi, Mi) with or without 500 nM gefitinib (EGFRi, Ei) for 72H. Data presented as in E. (H) Western blots of NCI-H508 cells treated with DMSO or 2.5 nM encorafenib alone or in combination with in combination with 10 μg/ml cetuximab (top) or 250 nM gefitinib (bottom) for 48H. (I) Relative gene expression of indicated genes in NCI-H508 cells treated with DMSO or 2.5 nM encorafenib (BRAFi) alone or in combination with 10 μg/ml cetuximab (EGFRi, cetux) for 48H. Data presented as in B. (J) Relative gene expression of indicated genes in NCI-H508 cells treated with DMSO or 2.5 nM encorafenib (BRAFi) alone or in combination with 250 nM gefitinib (EGFRi, gef) for 48H. Data presented as in B. (K) Immunofluorescence for β3-tubulin (B3T) in NCI-H508 cells treated as in I for 72H. Data presented as in E. (L) Normalized expression of *TGFBR2* in the indicated samples from the TCGA colon adenocarcinoma data. (M) T7 endonuclease 1 mismatch detection assay using primers that span the targeted regions for CRISPR knockout (KO) for Trp53 and Tgfbr2 in mock and TP (Trp53 + Tgfbr2) KO mouse colon CRC organoids. Gels are cropped. (N) and (O) Relative gene expression of the indicated genes in mock and TP KO mouse organoids. Data presented as in B. (P) Relative gene expression of the indicated genes in TP KO organoids treated with DMSO or 2.5 nM encorafenib (Bi) and 500 nM gefitinib (Ei) for 72H. Data presented as in B. (Q) Relative gene expression of the indicated genes in empty vector (EV) and NGN3 knockdown (KD) HT29 cells treated as in C for 48H and presented as in B. (R) T7 endonuclease 1 mismatch detection assay using primers that span

the targeted regions for CRISPR knockout (KO) for Ngn3 in mock and Ngn3 KO mouse colon TP KO CRC organoids. Ctrl is a control template used as positive control for T7 activity. Gels are cropped. (S) Relative gene expression of the indicated genes in scramble KO (Scr) and NGN3 KO TP KO organoids treated as in P and presented as in B. Significance was determined by ordinary one-way ANOVA with Tukey pairwise multiple comparison testing (B,C,D,E,F,G,I,J,K,L,Q,S) and Student's t-test (N,O,P). *P≤ 0.05, ** P ≤ 0.01, *** P ≤ 0.001, **** P ≤ 0.0001 relative to DMSO or Mock control. $^{\#}$P≤ 0.05, $^{\#\#\#}$P≤ 0.001, $^{\#\#\#\#}$P≤ 0.0001 relative to EV Bi or Scr Bi+Ei.

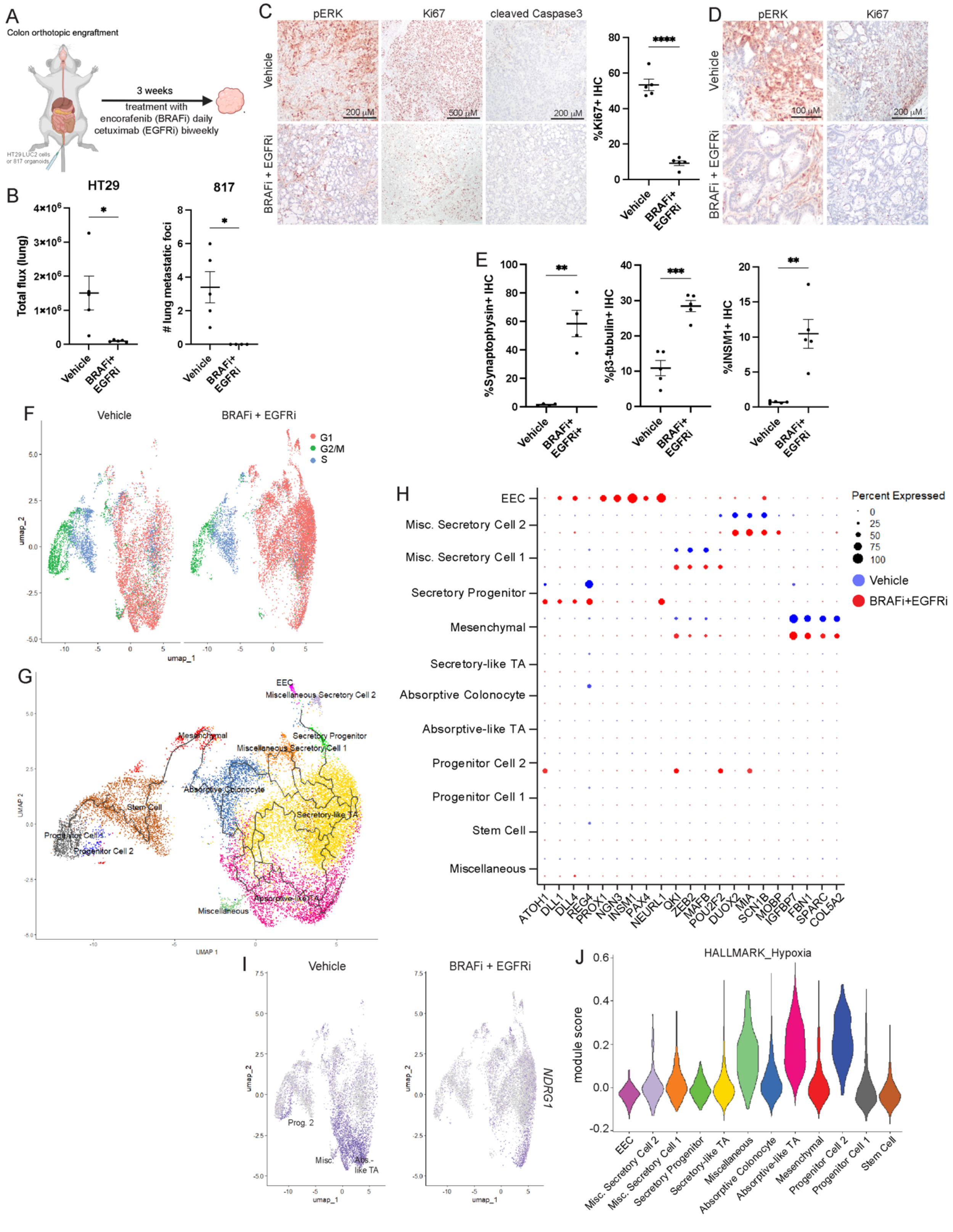

Supplementary Figure S2. Effect of BRAFi plus EGFRi on tumor cell type composition in *BRAF*^{V600E} CRC. (A) Diagram of HT29 and 817 colon orthotopic model and treatment. (B) Total

ex vivo flux of lungs or number of metastatic foci in lungs from mice with colon orthoptic HT29 or 817 tumors, respectively. Each dot represents the signal from lung from one mouse. Lines represent mean +/- SEM. (C&D) Representative IHC for indicated proteins in (C) HT29 or (D) 817 tumor sections from mice treated as indicated. (E) Scoring of HT29 tumor IHC staining of indicated proteins. Each point represents the scoring for one tumor. Lines represent mean -/+ SEM. (F&G) UMAP dot plots detailing predicted (F) cell cycle phase and (G) trajectory analysis of 817 scRNAseq samples. (H) Dot plot showing marker gene expression in vehicle and BRAFi+EGFRi treated samples across all annotated cell types. The size of the dot is proportional to the percentage of cells that express a given gene, and the color scale indicates the average scaled gene expression within the specific cell population. Blue and red dots are expression levels in cells from vehicle and BRAFi+EGFRi tumors, respectively. (I) UMAP dot plots of normalized expression values of *NDRG1* separated by treatment. (J) Module scores for the HALLMARK_hypoxia gene set for each cluster in the 817 scRNAseq data.

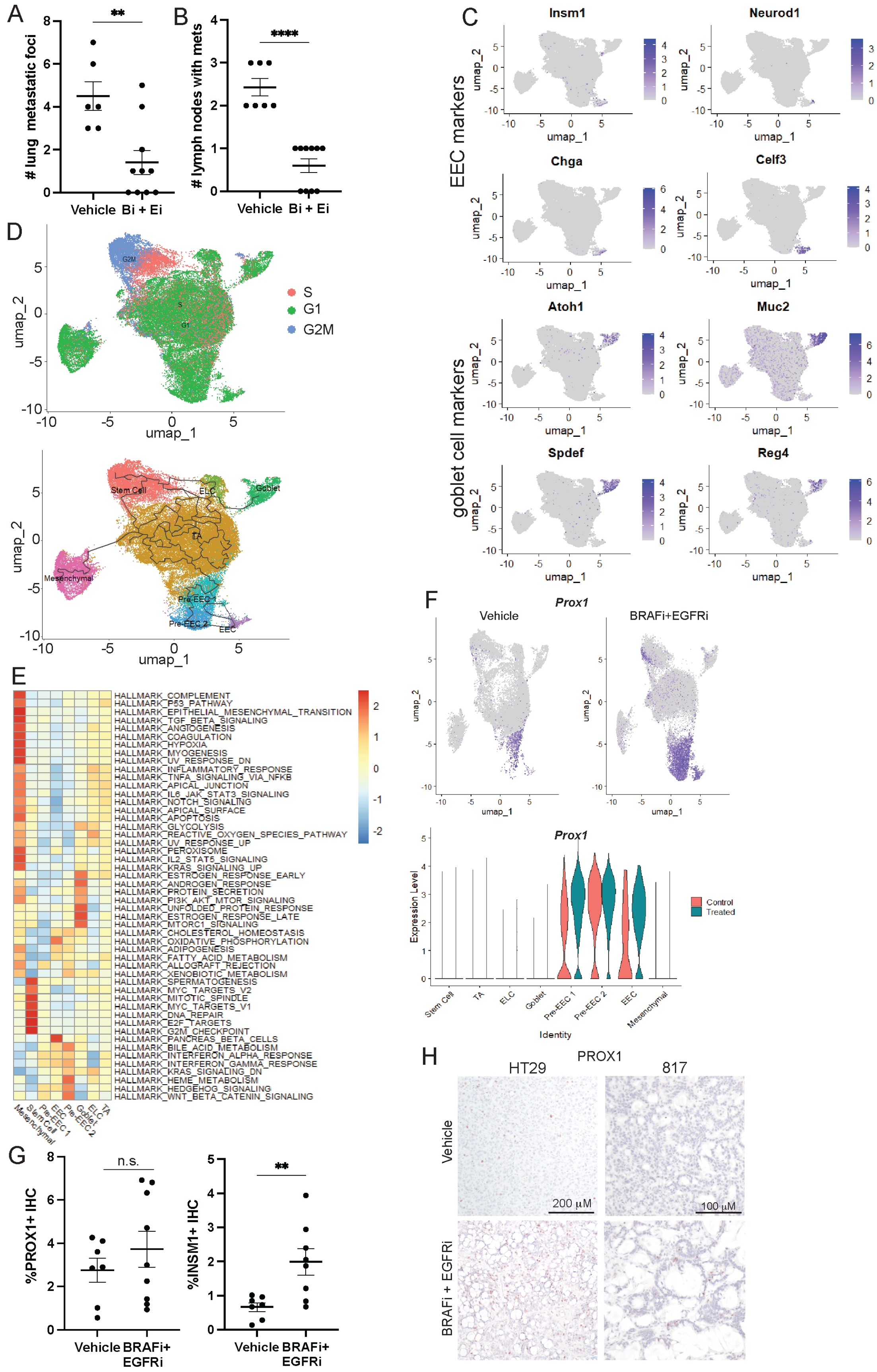
A
lung metastatic foci
**
Vehicle Bi + Ei
B
lymph nodes with mets
****
Vehicle Bi + Ei
C
EEC markers
goblet cell markers
Insm1
Neurod1
Chga
Celf3
Atoh1
Muc2
Spdef
Reg4
umap_1
umap_2
D
S
G1
G2M
Stem Cell
ELC
Goblet
TA
Mesenchymal
Pre-EEC 1
Pre-EEC 2
EEC
E
HALLMARK_COMPLEMENT
HALLMARK_P53_PATHWAY
HALLMARK_EPITHELIAL_MESENCHYMAL_TRANSITION
HALLMARK_TGF_BETA_SIGNALING
HALLMARK_ANGIOGENESIS
HALLMARK_COAGULATION
HALLMARK_HYPOXIA
HALLMARK_MYOGENESIS
HALLMARK_UV_RESPONSE_DN
HALLMARK_INFLAMMATORY_RESPONSE
HALLMARK_TNFA_SIGNALING_VIA_NFKB
HALLMARK_APICAL_JUNCTION
HALLMARK_IL6_JAK_STAT3_SIGNALING
HALLMARK_NOTCH_SIGNALING
HALLMARK_APICAL_SURFACE
HALLMARK_APOPTOSIS
HALLMARK_GLYCOLYSIS
HALLMARK_REACTIVE_OXYGEN_SPECIES_PATHWAY
HALLMARK_UV_RESPONSE_UP
HALLMARK_PEROXISOME
HALLMARK_IL2_STAT5_SIGNALING
HALLMARK_KRAS_SIGNALING_UP
HALLMARK_ESTROGEN_RESPONSE_EARLY
HALLMARK_ANDROGEN_RESPONSE
HALLMARK_PROTEIN_SECRETION
HALLMARK_PI3K_AKT_MTOR_SIGNALING
HALLMARK_UNFOLDED_PROTEIN_RESPONSE
HALLMARK_ESTROGEN_RESPONSE_LATE
HALLMARK_MTORC1_SIGNALING
HALLMARK_CHOLESTEROL_HOMEOSTASIS
HALLMARK_OXIDATIVE_PHOSPHORYLATION
HALLMARK_ADIPOGENESIS
HALLMARK_FATTY_ACID_METABOLISM
HALLMARK_ALLOGRAFT_REJECTION
HALLMARK_XENOBIOTIC_METABOLISM
HALLMARK_SPERMATOGENESIS
HALLMARK_MYC_TARGETS_V2
HALLMARK_MITOTIC_SPINDLE
HALLMARK_MYC_TARGETS_V1
HALLMARK_DNA_REPAIR
HALLMARK_E2F_TARGETS
HALLMARK_G2M_CHECKPOINT
HALLMARK_PANCREAS_BETA_CELLS
HALLMARK_BILE_ACID_METABOLISM
HALLMARK_INTERFERON_ALPHA_RESPONSE
HALLMARK_INTERFERON_GAMMA_RESPONSE
HALLMARK_KRAS_SIGNALING_DN
HALLMARK_HEME_METABOLISM
HALLMARK_HEDGEHOG_SIGNALING
HALLMARK_WNT_BETA_CATENIN_SIGNALING
Mesenchymal
Stem Cell
Pre-EEC1
EEC
Pre-EEC 2
Goblet
ELC
TA
F
Prox1
Vehicle
BRAFi+EGFRi
Expression Level
Control
Treated
Stem Cell
TA
ELC
Goblet
Pre-EEC 1
Pre-EEC 2
EEC
Mesenchymal
Identity
G
%PROX1+ IHC
n.s.
Vehicle BRAFi+ EGFRi
%INSM1+ IHC
**
Vehicle BRAFi+ EGFRi
H
PROX1
HT29
817
Vehicle
BRAFi + EGFRi
200 µM
100 µM

Supplementary Figure S3. Effect of BRAFi plus EGFRi on tumor cell type composition in a syngeneic model of *BRAF*^{V600E} CRC. (A) Number of metastatic foci in lungs from mice with colon orthotopic TP KO tumors treated with encorafenib (Bi) or gefitinib (Ei) as determined by IVIS imaging. Each dot represents the lung from one mouse. Lines represent mean +/- SEM. (B) Number of lymph nodes with metastases per mouse as determined by IVIS imaging. Each dot represents the number of positive lymph nodes in one mouse. Lines represent mean +/- SEM. (C) UMAP dot plots of normalized expression values of marker genes representative of EEC and goblet cell populations in TP KO scRNAseq samples. (D) UMAP dot plots detailing predicted cell cycle phase and trajectory analysis of TP KO scRNAseq samples. (E) Heatmap of enrichment scores for Hallmark gene sets by cell cluster in the TP KO scRNAseq data. (F) UMAP dot plot and violin plot of normalized *Prox1* expression in TP KO scRNAseq data separated by treatment type. (G) %PROX1 positive IHC staining of TP KO tumors as quantified by ImageJ. Each dot represents an individual tumor. Lines represent mean -/+ SEM. (H) Representative PROX1 IHC in HT29 and 817 colon orthotopic tumors. Significance was determined by Student's t-test. *P≤ 0.05, ** P ≤ 0.01, *** P ≤ 0.001, **** P ≤ 0.0001, n.s. – not significant.

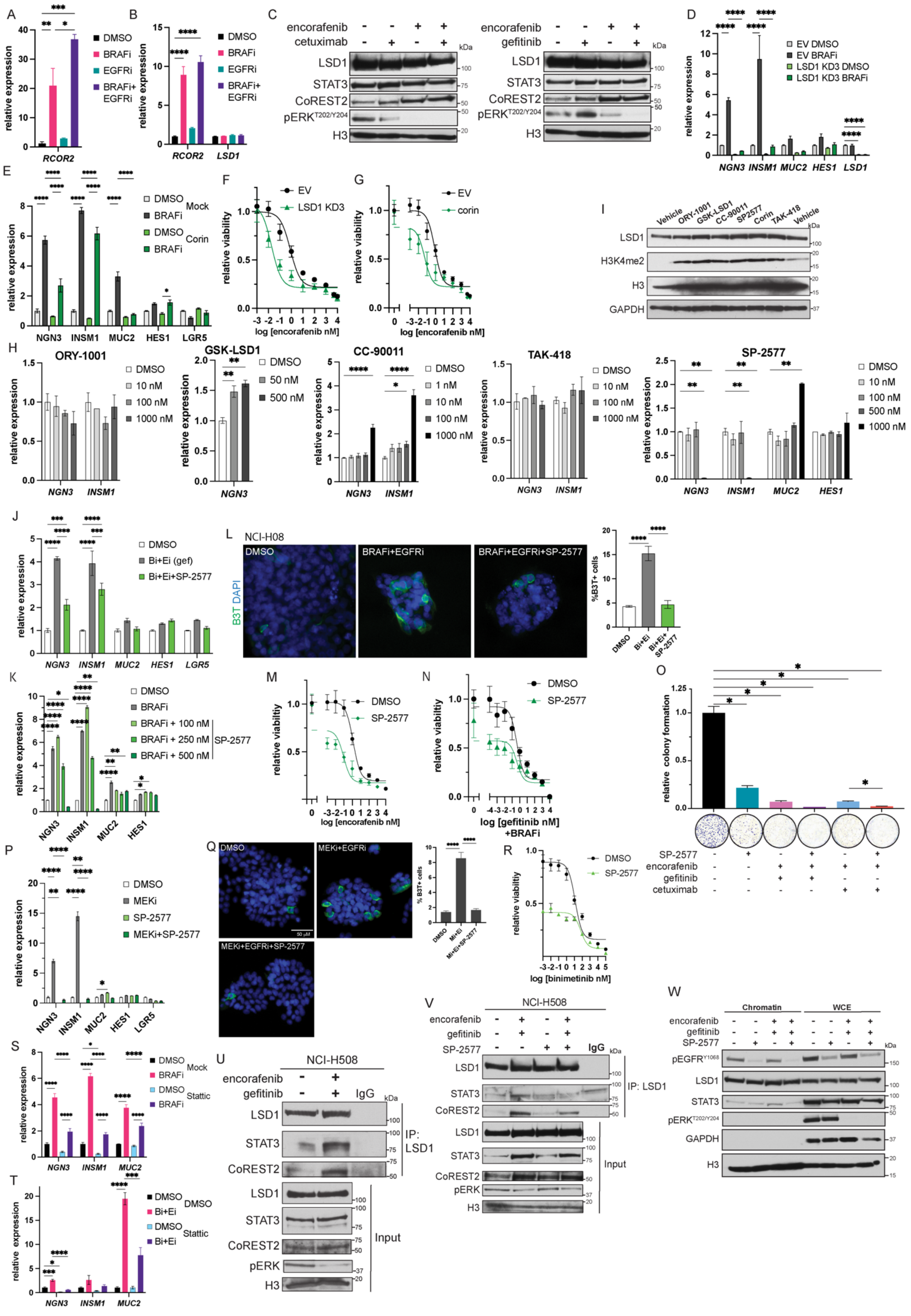

A
relative expression
RCOR2
DMSO
BRAFi
EGFRi
BRAFi+ EGFRi
B
RCOR2
LSD1
C
encorafenib
cetuximab
gefitinib
kDa
LSD1
STAT3
CoREST2
pERK T202/Y204
H3
D
EV DMSO
EV BRAFi
LSD1 KD3 DMSO
LSD1 KD3 BRAFi
NGN3
INSM1
MUC2
HES1
LSD1
E
Mock
Corin
LGR5
F
EV
LSD1 KD3
relative viability
log [encorafenib nM]
G
corin
I
Vehicle
ORY-1001
GSK-LSD1
CC-90011
SP2577
Corin
TAK-418
H3K4me2
GAPDH
H
ORY-1001
GSK-LSD1
CC-90011
TAK-418
SP-2577
J
Bi+Ei (gef)
Bi+Ei+SP-2577
L
NCI-H08
BRAFi+EGFRi
BRAFi+EGFRi+SP-2577
B3T DAPI
%B3T+ cells
K
BRAFi + 100 nM
BRAFi + 250 nM
BRAFi + 500 nM
SP-2577
M
SP-2577
N
log [gefitinib nM] +BRAFi
O
relative colony formation
encorafenib
gefitinib
cetuximab
P
MEKi
MEKi+SP-2577
Q
MEKi+EGFRi
MEKi+EGFRi+SP-2577
% B3T+ cells
R
log [binimetinib nM]
S
Stattic
T
Bi+Ei
U
NCI-H508
IgG
IP: LSD1
Input
pERK
V
W
Chromatin
WCE
pEGFR Y1068

Supplementary Figure S4. LSD1 depletion blocks MAPK pathway inhibition-induced increase in EECs. (A) Relative gene expression of indicated genes in HT29 cells treated with DMSO or 2.5 nM encorafenib (BRAFi) and 20 μg/ml cetuximab (EGFRi) for 48H. Gene expression was normalized to the housekeeping gene *RHOA* and then to the DMSO treated cells. Graph represents mean +/- SEM. N=3. (B) Relative gene expression of indicated genes in HT29 cells treated with DMSO or 2.5 nM encorafenib (BRAFi) and/or 500 nM gefitinib (EGFRi) for 48H. Data presented as in A. (C) Western blot of HT29 cells treated as indicated and as in A and B. (D) Relative gene expression in empty vector (EV) and LSD1 knockdown (KD) HT29 cells treated with DMSO or 2.5 nM encorafenib (BRAFi) for 48H. Data presented as in A. LSD1 KD3 is performed using distinct shRNA from those used in primary Figure 1A and 1B. (E) Relative gene expression of indicated genes in HT29 cells treated with DMSO or 2.5 nM encorafenib with or without 50 nM corin for 48H. Data is normalized and presented as in A. (F) Encorafenib dose response curve of EV and LSD1 HT29 cells treated for 72H. Viability was normalized to each group's respective non-encorafenib treated cells. (G) Encorafenib dose response curve of HT29 cells treated with 50 nM corin for 72H. Normalized as in F. (H) Relative gene expression in HT29 cells treated with the indicated concentrations of the indicated LSD1 inhibitors. Graph represents mean +/- SEM. N=3. (I) Western blot of cell lysates prepared from HT29 cells treated with 10 nM of the indicated LSD1 inhibitors. (J) Relative gene expression of indicated genes in NCI-H508 cells treated with DMSO or 2.5 nM encorafenib plus 250 nM gefitinib (EGFRi, Ei, gef) for with or without 1 μM SP-2577 for 48H. Data is normalized and presented as in A. (K) Relative gene expression of indicated genes in HT29 cells treated with DMSO or 2.5 nM encorafenib with or without the indicated concentration of SP-2577 for 48H. Data is normalized and presented as in A. (L) Immunofluorescence for β3-tubulin in NCI-H508 cells treated as in J for 72H. Graph is the %β3-tubulin+ cells of the total number of cells per field. N=3. (M) Encorafenib dose response curve of HT29 cells treated with 500 nM SP-2577 for 72H. Normalized as in F. (N) Gefitinib dose response curve of NCI-H508 cells treated with 2.5 nM encorafenib (BRAFi) and DMSO or 500 nM SP-2577 for 72H. Normalized as in F. (O) Colony formation assay of HT29 cells treated with DMSO or 2.5 nM encorafenib and 500 nM gefitinib or 20 μg/ml cetuximab with or without 1 μM SP-2577 for 6 days. Data is normalized to DMSO treated cells. Graph represents mean +/- SEM. N=3. (P) Relative gene expression of indicated genes in HT29 cells treated with DMSO or 20 nM binimetinib (MEKi) with or without 500 nM SP-2577 for 48H. Data is normalized and presented as in A. (Q) Immunofluorescence for β3-tubulin in HT29 cells treated with DMSO or 20 nM binimetinib (MEKi; Mi) and 500 nM gefitinib (EGFRi, Ei) with or without 500 nM SP-2577for 72H. Data presented as in L. (R) Binimetinib dose response curve of HT29 cells treated with 500 nM SP-2577 for 72H. Viability was normalized to cells treated with DMSO only. (S) Relative gene expression of indicated genes in HT29 cells treated with DMSO or 2.5 nM encorafenib (BRAFi) with or without 2 μM Stattic (STAT3i) for 48H. Data is normalized and presented as in A. (T) Relative gene expression of indicated genes in NCI-H508 cells treated with DMSO or 2.5 nM encorafenib (Bi) and 250 nM gefitinib (Ei) with or without 5 μM Stattic (STAT3i) for 48H. Data is normalized and presented as in A. (U) LSD1 coIP in nuclear lysates prepared from NCI-H508 cells treated with DMSO or 2.5 nM encorafenib plus 250 nM gefitinib for 4H. (V) LSD1 coIP in nuclear lysates prepared from NCI-H508 cells pre-treated with DMSO or 1 μM SP-2577 for 24H prior to treatment with DMSO or 1 μM SP-2577 with or without 2.5nM encorafenib plus 250 nM geftinib for 4H. (W) Western blot of chromatin and whole cell extract (WCE) fractions prepared from HT29 cells pre-treated with DMSO or 1 μM SP-2577 for 24H prior to treatment of DMSO or 1 μM SP-2577 with or without 2.5 nM encorafenib plus 500 nM gefitinib for 4H. Significance was determined by ordinary one-way ANOVA with Tukey pairwise multiple comparison testing for all panels except O, which was analyzed by Brown-Forsythe and Welch ANOVA test with Dunnett's T3 multiple comparisons test. *$P \leq 0.05$, ** $P \leq 0.01$, *** $P \leq 0.001$, **** $P \leq 0.0001$.

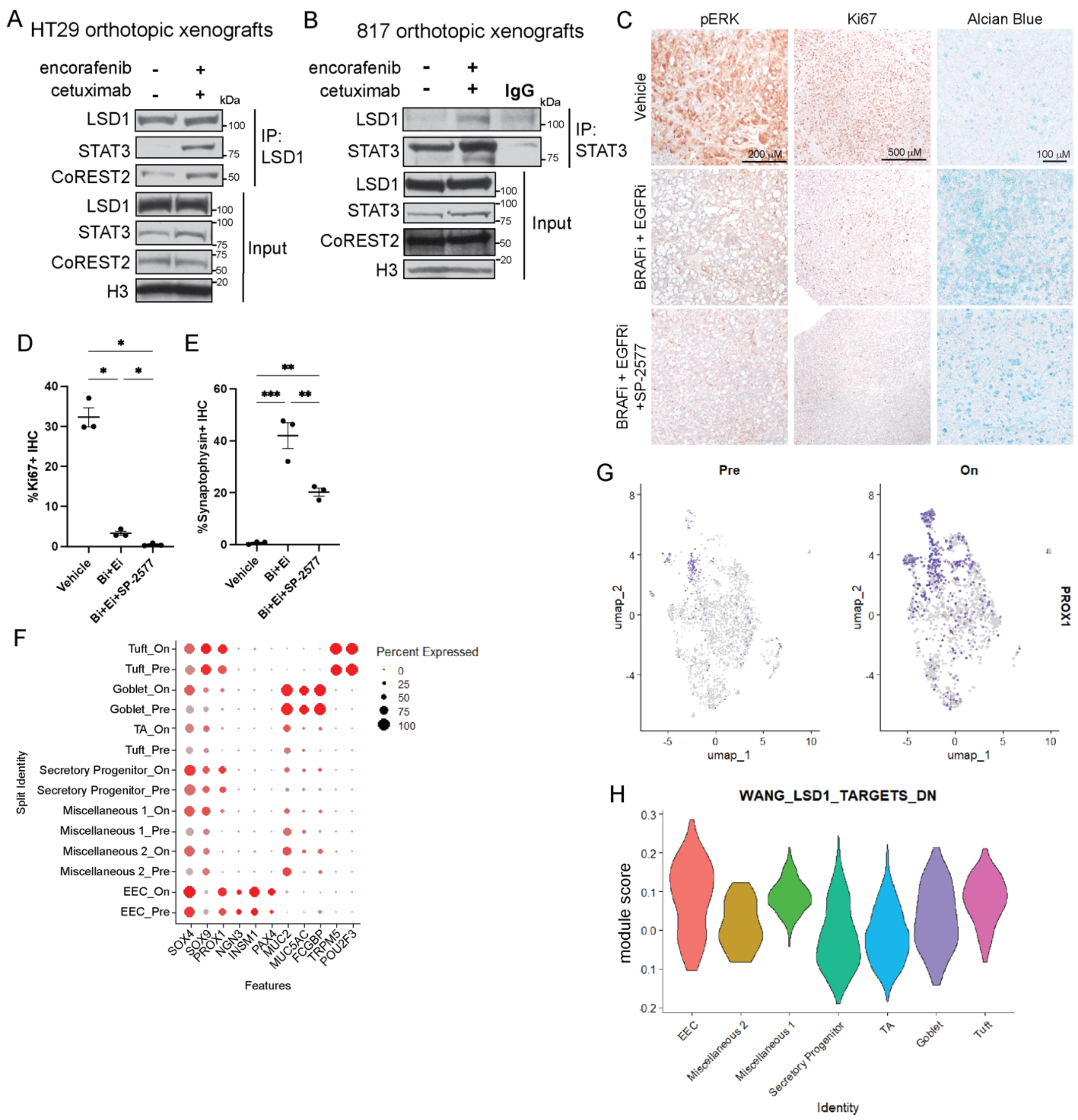

Supplementary Figure S5. Epithelial cancer cell types present in patient samples of *BRAF^{V600E}* CRC. (A) LSD1 coIP in nuclear lysates prepared from HT29 colon orthotopic tumors from vehicle or encorafenib + cetuximab treated NSG mice. The same tumor lysates were also used in the experiment in 5G, so the input blots are the same. (B) STAT3 coIP in nuclear lysates prepared from 817 colon orthotopic tumors from vehicle or encorafenib + cetuximab treated NSG mice. All images of western blots are cropped. (C) Representative IHC for indicated proteins in HT29 orthotopic tumor sections from mice treated as indicated. (D and E) Scoring of IHC staining of indicated proteins. Each point represents the scoring for one tumor. Lines represent mean -/+ SEM. (F) Dot plot showing marker gene expression in pretreatment and on treatment samples across all annotated cell types. The size of the dot is proportional to the percentage of cells that express a given gene, and the color scale indicates the average scaled gene expression within the specific cell population. (G) UMAP dot plot of normalized *PROX1* expression in samples from patient with *BRAF^{V600E}* CRC scRNAseq data separated by

treatment type. (H) Module scores for the Wang LSD1 Targets Down gene set for each cluster in the scRNAseq data from patients with *BRAF*^{V600E} CRC.
